# A comprehensive approach to elucidating the pathophysiology of kidney fibrosis based on extracellular vesicle proteomics

**DOI:** 10.3389/fphys.2026.1786999

**Published:** 2026-05-29

**Authors:** Yaerim Kim, Kyuhyeon Kim, Hong-Beom Park, Sunwha Lee, Mi-yeon Yu, Young Joo Kim, Soo Bin Choi, Woo Yeong Park, Kyubok Jin, Dong Ki Kim, Yon Su Kim, Dohyun Han, Seung Hee Yang

**Affiliations:** 1 Department of Internal Medicine, Keimyung University School of Medicine, Daegu, Republic of Korea; 2 Biomedical Research Institute, Seoul National University, Seoul, Republic of Korea; 3 Division of Nephrology, Department of Internal Medicine, Kangwon National University Hospital, Chuncheon, Republic of Korea; 4 Division of Nephrology, Department of Internal Medicine, Hanyang University Guri Hospital, Guri, Republic of Korea; 5 Department of Internal Medicine, Seoul National University College of Medicine, Seoul, Republic of Korea; 6 Kidney Research Institute, Seoul National University College of Medicine, Seoul, Republic of Korea; 7 Proteomics Core Facility, Biomedical Research Institute, Seoul National University Hospital, Seoul, Republic of Korea

**Keywords:** chronic kidney disease, extracellular vesicle, proteomics, transglutaminase 2, tubulointerstitial fibrosis

## Abstract

**Introduction:**

Transglutaminase 2 (TG2) plays a profibrotic role in chronic kidney disease (CKD), but its role in the exosomal proteome remains unexplored. Here, we aimed to evaluate biological processes specifically involved in CKD progression through exosomal proteomic profiling following TG2 inhibition.

**Materials and methods:**

Human proximal tubular epithelial cells (hPTECs) were treated with recombinant transforming growth factor-β (rTGF-β) to induce fibrosis and cysteamine to inhibit TG2. A unilateral ureteral obstruction (UUO) mouse model was used for *in vivo* validation. EVs were isolated and analyzed using LC-MS/MS analysis. Bioinformatics tools, including enrichGO and STRING, were used to identify key biological pathways and protein interactions. Findings were validated in the UUO mouse model.

**Results:**

TG2 inhibition attenuated the expression of fibrosis- and inflammation-associated proteins in hPTECs and fibroblasts. EV proteomic analysis revealed distinct protein expression patterns following rTGF-β treatment and TG2 inhibition. Altered proteins were primarily extracellular matrix components such as connective tissue growth factor, IGF-binding protein, laminin, plasminogen activator inhibitor, periostin, and collagen.

**Conclusions:**

TG2 inhibition modulated EV-associated proteins involved in fibrosis and inflammation, highlighting its therapeutic potential in CKD. Identifying reversible fibrosis-related factors may provide new targets for CKD treatment.

## Introduction

Chronic kidney disease (CKD) is a major global public health concern, thus understanding its pathophysiology is crucial. Among various pathological processes, tubulointerstitial fibrosis is tightly linked to the development of CKD (Tampe and Zeisberg, 2014; Tang et al., 2019). The histopathological characteristics of tubulointerstitial fibrosis are defined by excessive extracellular matrix (ECM) accumulation, which is associated with inflammatory cell infiltration, damage of tubular epithelial cells, and profibrotic cytokine production with activation of transforming growth factor β (TGF-β) signaling pathway.

Sustained injury to tubular epithelial cells initiates the release of pro-inflammatory cytokines and chemokines, leading to recruitment and activation of immune cells and amplification of the inflammatory response (Qi and Yang, 2018; Black et al., 2019; Reck et al., 2025). This inflammatory milieu promotes activation of profibrotic signaling pathways, particularly the TGF-β/Smad axis, which drives myofibroblast differentiation and excessive production of ECM components such as type I and III collagens (Huang et al., 2023; Lathan, 2024). In parallel, imbalance between matrix metalloproteinases and their inhibitors results in impaired ECM degradation, further accelerating matrix accumulation (Bülow and Boor, 2019; La Russa et al., 2024). Progressive capillary rarefaction and tissue hypoxia subsequently contribute to tubular atrophy and irreversible loss of renal function (Schelling, 2016; Tanabe et al., 2020).

The human transglutaminase 2 (TG2) is a multifunctional enzyme whose activities are tightly regulated by intracellular Ca²^+^ levels. Under basal conditions, TG2 predominantly adopts a closed, GTP-bound conformation and functions as a GTPase (Iismaa et al., 1997), protein disulfide isomerase (Hasegawa et al., 2003), and serine/threonine kinase (Mishra and Murphy, 2004). In contrast, elevations in intracellular Ca²^+^ induce a conformational switch to an open state, enabling its transamidase activity, which catalyzes the formation of ϵ-(γ-glutamyl) lysine isopeptide bonds between protein substrates (Fesus and Piacentini, 2002; Lorand and Graham, 2003). This Ca²^+^-dependent activation is particularly relevant under pathological conditions such as tissue injury and inflammation, where intracellular Ca²^+^ levels are increased. TG2 is widely distributed across cellular compartments, including the nucleus, mitochondria, cytoplasm, cell membrane, and ECM, and plays a crucial role in the pathogenesis of fibrosis. TG2 acts as a central mediator of fibrotic processes by promoting ECM stabilization and amplification of profibrotic signaling (Prat-Duran et al., 2021). TG2 crosslinks key ECM components, such as fibronectin and collagen type I, leading to resistance against proteolytic degradation. TG2 also forms covalent links between fibrillin-1 and latent TGF-β complexes necessary for activating TGF-β. Once activated, TGF-β binds to the TGF-β receptors in the cell membrane and triggers morphological transitions and increased ECM accumulation via the canonical SMAD signaling pathway (Ju et al., 2023). Through these mechanisms, TG2 enhances myofibroblast activation and persistent ECM accumulation, thereby driving the progression of kidney fibrosis. Notably, our recent findings demonstrate that TG2 suppression using cysteamine, a competitive TG2 inhibitor already approved for treating cystinosis, protects against kidney fibrosis by reducing ECM accumulation, apoptosis, and inflammatory responses (Moon et al., 2022).

Extracellular vesicles (EVs) are nano-sized biovesicles that are released in abundance from most cell types, including exosomes, microvesicles, and other subtypes. Previously, these particles were considered cellular waste attributed to cell damage or by-products of cell homeostasis with no significant role in neighboring cells. However, recent studies have shown that these extracellular vesicles are functional vehicles that transport complex cargo, including proteins and nucleic acids (Valadi et al., 2007; Simpson et al., 2009; Waldenström et al., 2012). EVs reflect their cellular origin and physiological states, rendering them powerful biomarkers for monitoring disease progression and treatment responses. In the context of kidney diseases, EVs serve not only as real-time indicators of kidney injury but also as active mediators of disease progression (Grange and Bussolati, 2022; Li et al., 2024). Indeed, EVs derived from fibroblasts and tubular epithelial cells have been shown to carry miRNA cargo that promotes apoptosis, M1 macrophage activation, and subsequent renal fibrosis (Zhang et al., 2019; Erdbrügger et al., 2021). These findings highlight the dual role of EVs as both biomarkers and functional drivers of kidney fibrosis. Interestingly, TG2 has been shown to impact EV biogenesis and its cargo composition by interacting with the endosomal sorting complex required for transport (ESCRT) protein machinery in mouse embryonic fibroblasts under cellular stress induced by proteasome inhibition (Diaz-Hidalgo et al., 2016). However, the impact of TG2 in the EV proteome in the context of kidney fibrosis has not yet been fully explored. Investigating the proteomic changes in EVs following TG2 inhibition using cysteamine in human primary tubular epithelial cells (hPTECs) could help elucidate how TG2 modulates ECM remodeling, inflammatory responses, and apoptosis, which are central to kidney fibrosis progression.

Despite the established roles of TG2 in TGF-β–mediated fibrosis and the growing recognition of EVs as key mediators of intercellular communication, it remains unclear how TG2 influences the EV-associated proteomic landscape during fibrotic processes. We hypothesized that TG2 inhibition by cysteamine remodels EV cargo away from a profibrotic signature. In this study, we investigated the effects of TG2 inhibition on EV-derived proteomic changes under TGF-β–induced fibrotic conditions and sought to identify key pathways and proteins associated with fibrosis progression and reversibility.

## Materials and methods

### Sample acquisition and primary human proximal tubular epithelial cell culture

Sample acquisition and all experiments involving hPTECs were conducted under the strict guidelines approved by the Institutional Review Board of Seoul National University Hospital (IRB no. 1002-045-309), with informed consent obtained from all participants prior to participation. Kidney tissues were obtained from patients with renal cell carcinoma undergoing surgical nephrectomy. Normal cortical kidney tissue was extracted from a section completely separated from the malignant tissue. hPTECs were cultured as described previously (Kim et al., 2021a).

hPTECs were treated with 2 ng/mL rTGF-β (R&D Systems, Minneapolis, MN, USA; Cat# 240–B) and 0.5, 1, 2, or 4 mM cysteamine (Sigma-Aldrich, St. Louis, MO, USA; Cat# 30070) in Renal Epithelial Basal Medium (Lonza, Basel, Switzerland; Cat# CC-3190) with 3% exosome-depleted FBS (Gibco, Waltham, MA, USA; Cat# A2720801). The cells were incubated at 37 °C and 5% CO_2_ for 48 h. NIH3T3 cells (ATCC: CRL-1658) were maintained in DMEM/F12 (Biowest, Riverside, MO, USA; Cat# L0092) supplemented with 10% FBS (Gibco, Cat# A4766801) and 1% 100X penicillin-streptomycin (Gibco, Cat# 15140-122). NIH3T3 cells were treated with 2 ng/mL of rTGF-β and 0.5, 1, or 2 mM cysteamine for 24 h.

### Isolation of EVs

The medium of treated hPTECs was collected and centrifuged at 2,000 × *g* for 30 min at 4 °C to remove dead cells and cell debris. The remaining conditioned medium was concentrated using a 30 kDa MWCO centrifugal filter (Millipore Sigma, Burlington, MA, USA; Cat# UFC9030) at 4,000 × *g* for 20 min at 18 °C. The concentrate was incubated overnight at 4 °C with an equal volume of the Total Exosome Isolation Kit (Thermo Fisher, Waltham, MA, USA; Cat# 4478359) to the concentrate to aggregate EVs. The mixture was centrifuged at 10,000 × *g* for 1 h at 4 °C the next day, and the supernatant was carefully aspirated. The remaining EV pellets were used for downstream analyses.

### Nanoparticle tracking analysis

Nanosight NS300 (Malvern Panalytical, Worcestershire, England) and NTA 3.4 Build 3.4.003 software were used to measure the hydrodynamic diameters of nanoparticles. EV pellets were diluted with 1X PBS until a minimum of 10 particle count per frame was obtained. sCMOS red laser camera was used at 11 camera level, 25 FPS, 8 gain, and 3 threshold at 22 °C for all samples.

### *In vivo* experiments

Kidney fibrosis was induced by unilateral ureteral obstruction (UUO) in C57BL/6 mice (male, 7 weeks old, 25 g) purchased from Koatech (Seoul, South Korea), a well-established and reproducible model of tubulointerstitial fibrosis (Okamura et al., 2014). Xylazine (Rompun; 10 mg/kg of body weight; Bayer, Mississauga, ON, Canada; Cat# 17033-099-05) was administered intraperitoneally. A 1:1 mixture of tiletamine and zolazepam (Zoletil™; 30 mg/kg body weight; Virbac, Carroll, France; Cat# 51311-718-05) was administered. The left kidney was approached via a flank incision, and the ureter was ligated using 5–0 silk. Heat pads were used to maintain a temperature of 37 °C for mice, and warmed PBS at 37 °C was administered intraperitoneally to prevent dehydration. Identical surgical procedures were performed in sham-operated mice without ureter ligation. After 3, 7, and 14 days, mice were euthanized by CO_2_ inhalation in a chamber for 30 minutes, and their kidneys were immediately harvested for further analysis. All animal experiments were conducted strictly using relevant guidelines and regulations approved by the Seoul National University Hospital Institutional Animal Care and Use Committee (permit number: 20-0009-S1A0). The study was reported in accordance with the Animal Research: Reporting of *In Vivo* Experiments (ARRIVE) guidelines and in accordance with relevant guidelines and regulations.

### Western blot

Proteins were extracted from hPTECs, NIH3T3 cells, EVs, and mouse kidneys for western blotting. Cultured cells were washed with 1X PBS and then were treated with lysis buffer (RIPA buffer (Millipore Sigma; Cat# R0278), protease inhibitor cocktail (Millipore Sigma; Cat# P8340), EDTA (Millipore Sigma; Cat# E9884), and PMSF (Millipore Sigma; Cat# PMSF-RO)) for 30 min at 4 °C. EV pellets were directly treated with lysis buffer for 30 min at 4 °C, followed by ultrasonication for 30 s. The supernatant was carefully collected after being centrifuged at 15,000 × *g* for 10 min at 4 °C. UUO mouse kidney tissues were harvested on days 3, 7, and 14. Kidneys were sliced and treated with lysis buffer. Samples were homogenized and centrifuged twice at 15,000 × *g* for 10 min at 4 °C, carefully collecting the supernatant at each step. Protein concentrations for all sample types were measured using the BCA assay (Thermo Fisher Scientific; Cat# 23225), and the lysates were mixed with 5X Laemmli buffer (Biosesang, Gyeonggi-do, South Korea; Cat# SF2002-110-00) and lysis buffer to the appropriate concentrations.

The extracted proteins were separated with gels of varying polyacrylamide concentrations (6–15%) and transferred onto Immobilon-FL 0.45 μM polyvinylidene difluoride membranes (Millipore Sigma; Cat# IPFL00010). Primary antibodies against β-actin (Sigma Aldrich; Cat# A1978), fibronectin (Abcam, Cambridge, MA, USA; Cat# ab2413), collagen type I-α1 (Santa Cruz Biotechnology, Dallas, TX, USA; Cat# sc293182), collagen type IV (Abcam; Cat# ab6586), periostin (Abcam; Cat# ab152099), α-smooth muscle actin (Abcam; Cat# ab32575), P21 (Santa Cruz Biotechnology; Cat# sc6246), CD63 (Thermo Fisher; Cat# PA5-92370), CD81 (Santa Cruz Biotechnology; Cat# sc166029), calnexin (Proteintech, Chicago, IL, USA; Cat# 66903-1-ig), tumor necrosis factor α (Abcam; Cat# ab66579), and integrin α5 (Abcam; Cat# ab179475), and TG2 (Abcam; Cat# ab421) were used (**Supplementary Table 1**). Horseradish peroxidase-conjugated rabbit IgG (Cell Signaling Technology, Danvers, MA, USA; Cat # 7076) and mouse IgG (Cell Signaling Technology; Cat # 7074) were used as secondary antibodies. SuperSignal West Femto Maximum Sensitivity Substrate (Thermo Fisher Scientific; Cat # 34094) was used as an HRP substrate. Chemiluminescent images were digitally captured using Image Quant LAS 4000 Mini (GE Healthcare, Chicago, IL, USA). The ImageJ software (National Institutes of Health, Bethesda, MD, USA) was used for image quantification. Full-length, uncropped western blot images corresponding to all western blot results are provided in **Supplementary Figure 1**.

### Fluorescence-activated cell sorting analysis

An Annexin V Apoptosis Detection Kit (BD Biosciences, Franklin Lakes, NJ, USA; Cat# 556547) was used to evaluate apoptosis and necrosis in hPTECs. Cells were harvested and washed with cold PBS, then resuspended in 100 μL of binding buffer at 5 × 10^5^ cells per tube. A total of 5 μL of FITC-conjugated Annexin V and 10 μL of propidium iodide was added to each tube, followed by incubation in the dark for 30 minutes at room temperature. BD FACSDiva™ (V8.0; BD Biosciences) was used to analyze the flow cytometry data.

### Immunohistochemistry

Mice kidneys were fixed overnight with 4% paraformaldehyde solution at 4 °C. Paraffinized mouse kidney sections (4 μm) were deparaffinized thrice with xylene for 5 min. The sections were then rehydrated using ethanol solutions in descending concentration orders of 100, 100, 95, 90, 80, and 70%, with each step lasting 5 min each. To visualize collagen deposition, the sections were stained with Sirius red (Abcam; Cat# ab150681). After blocking endogenous peroxidase activity with 3% hydrogen peroxide solution (Supelco, Bellefont, PA, USA; Cat# K54376509), sections were incubated five times in 10% citrate buffer at 95 °C, five minutes each. To observe the presence of TG2, sections were incubated in a Blocking Reagent (Agilent, Santa Clara, CA, USA; Cat# X0909) for 1 h at room temperature, followed by incubation with anti-TG2 antibody (Abcam; Cat# ab421) and goat anti-rabbit IgG (Vector Laboratories, Burlingame, CA, USA; Cat# AI-1000-1.5). Mayer’s hematoxylin (Sigma-Aldrich; Cat# MHS1) was used for counterstaining, and the sections were visualized under a light microscope (DFC-295; Leica, Mannheim, Germany).

### Sample preparation for interactome analysis

EV samples for mass spectrometry analysis were prepared as per the previously described method with some modifications (Lim et al., 2023). Briefly, the extraction buffer (2% sodium dodecyl sulfate, 5 mM Tris (2-carboxyethyl) phosphine, and 20 mM chloroacetamide in 50 mM ammonium bicarbonate) was added to the EV pellets. Digestion and desalting of the eluted proteins were performed using filter-aided sample preparation and StageTip methods, respectively, as previously described (Byun et al., 2023). The mixture was heated for 15 min at 95°C to reduce and alkylate the sample. The mixture was loaded onto a 30 K Amicon filter (Millipore, MA, USA; Cat# UFC9030). Buffer exchanges were performed with the UA solution (8M urea in 0.1 M Tris pH 8.5) via centrifugation at 14,000 × *g* for 15 min. Following an exchange of the buffer with 40 mM ammonium bicarbonate, protein digestion was performed overnight at 37 °C using trypsin/Lys-C Mix (Promega; Cat# V5071) at a 100:1 protein-to-protease ratio. All resulting peptides were acidified with 10% trifluoroacetic acid and desalted using a homemade C18 Styrenedivinylbenzene (SDB)-Reverse Phase Sulfonate (RPS) StageTip column. The peptides were eluted with elution buffer (40%, 60%, and 80% acetonitrile) into three fractions (Kim et al., 2021b). All eluted peptides were dried under a vacuum.

### Liquid chromatography-tandem MS analysis and data processing

LC-MS/MS analysis was performed using a Q-Exactive Plus mass spectrometer (Thermo Fisher Scientific, Waltham, MA, USA) coupled with an Ultimate 3000 RSLC nano system (Dionex, Sunnyvale, CA, USA), as previously described (Kim et al., 2024). Peptides were separated using a two-column system with a trap column (C18, 75 µm I.D x 2 cm length, 3 µm) and an analytic column (EASY-Spray C18, 75 µm I.D. × 50 cm length, 2 µm) with a 90 min gradient 5% - 35% ACN at a flow rate of 300 nl/min. MS measurements were conducted in the positive ion mode. For data-dependent acquisition, a survey scan was conducted over the 300–1,800 m/z range and the resolution was set at 60,000. The top 15 precursor ions were selected within an isolation window of 1.2 m/z. The MS/MS spectrum was acquired using a high-collision-dissociated-normalized collision energy of 30% and the resolution was set at 15,000.

The MS spectra were processed using MaxQuant software version 1.6.1.0 (Tyanova et al., 2016). MS/MS spectra were searched against the UniProt human protein sequence database (December 2014 release; 88,657 entries), including forward and reverse sequences and common contaminants. Primary searches were performed using a 6-ppm precursor ion tolerance for total protein level analysis. MS/MS ion tolerance was set to 20 ppm. Cysteine carbamidomethylation was used as a fixed modification. The N-acetylation of proteins and oxidation of methionine (M) were set as variable modifications. The enzyme specificity was set at full tryptic digestion. Peptides with a minimum length of six amino acids and up to two missed cleavages were considered search parameters. The false discovery rate (FDR) was set to 1% for peptide and protein identification. For label-free quantification, an intensity-based absolute quantification (iBAQ) algorithm (Schwanhäusser et al., 2011) was used as a part of the MaxQuant platform.

### Statistical analysis

Pre-processing and statistical analysis of the proteomic data were performed using the Perseus software (version 1.6.15.0) (Tyanova and Cox, 2018). Protein expression levels were estimated by determining logarithmic (log_2_(x)) iBAQ values. Valid values were filtered using proteins with a minimum of 70% quantification in at least one group. This threshold was applied to reduce false-positive identifications while retaining condition-specific proteins (Zhou et al., 2021). Missing values were imputed based on the normal distribution (width = 0.3, downshift = 1.8) to simulate low-abundance protein signals. Multiple comparison tests were performed using one-way ANOVA. Principal component analysis and hierarchical clustering were performed using the Perseus software. Two-tailed, unpaired Student’s t-tests were used for pairwise comparisons. Fold-change was calculated by determining the difference between the mean (log_2_(x)) iBAQ values of each condition group. The enrichGO R package and Enrichr database (https://maayanlab.cloud/Enrichr/enrich) were used for Gene Ontology (GO) enrichment analysis of the ANOVA clusters, which were further organized based on gene ratio values or FDR-adjusted p-values. Protein–protein interaction networks were generated using the STRING database (version 11.5; https://string-db.org/) and visualized in Cytoscape (version 3.10.0). A confidence score threshold of ≥0.700 (high confidence) was applied, and network edges were defined based on experimental evidence, curated databases, and co-expression data. All bar graphs are expressed as mean ± SEM, drawn using GraphPad Prism (version 10.2.3).

## Results

### Characteristics of EVs

**Figure 1:**
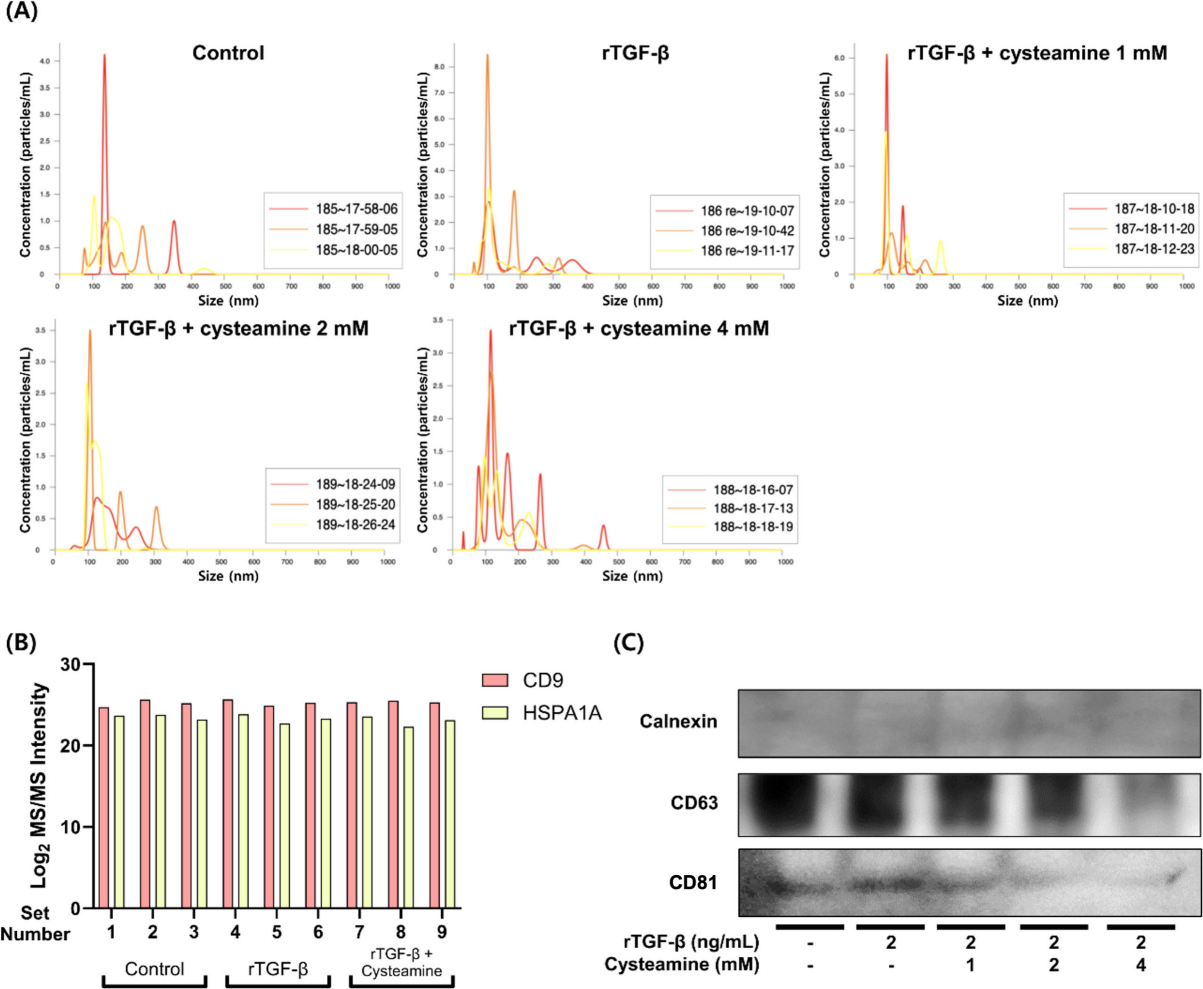
Comprehensive characterization of EVs isolated from human proximal tubular epithelial cells. **(A)** NTA results of the hydrodynamic diameter of nanoparticles (nm) and their Finite Track Length Adjustment (FTLA) concentration (10^6^ particles/mL), measured in triplicates for each condition. **(B)** Differential EV intensity patterns according to the three distinct conditions: control, treatment with 2 ng/mL of rTGF-β, and treatment with 2 ng/mL of rTGF-β with 2 mM cysteamine. Each condition was analyzed using three biological replicates. The color scheme denotes CD9 (red) and HSP70 (yellow). **(C)** Western blots of calnexin, CD63, and CD81 of EVs released by hPTECs treated with 2 ng/mL of rTGF-β and various concentrations (0, 1, 2, or 4 mM) of cysteamine for 48 h.

EVs derived from hPTECs were first characterized to confirm the quality and purity of the isolated vesicles. NTA results revealed that particles isolated from hPTEC media in all conditions were within the expected size range of exosomes (<150 nm) and microvesicles (200–500 nm) (Maas et al., 2017) (**Figure 1A**). LC-MS/MS analysis demonstrated the presence of proteins common to EVs, such as CD9 and HSP70, without statistical differences between the treatment conditions (**Figure 1B**). CD63 and CD81, tetraspanins also commonly found in EVs, were markedly expressed in western blots, in contrast to the absence of calnexin, a protein that binds to the endoplasmic reticulum (**Figure 1C**). These results confirm the successful isolation of EVs suitable for downstream proteomic analysis.

### Impact of TG2 on kidney fibrosis process

**Figure 2:**
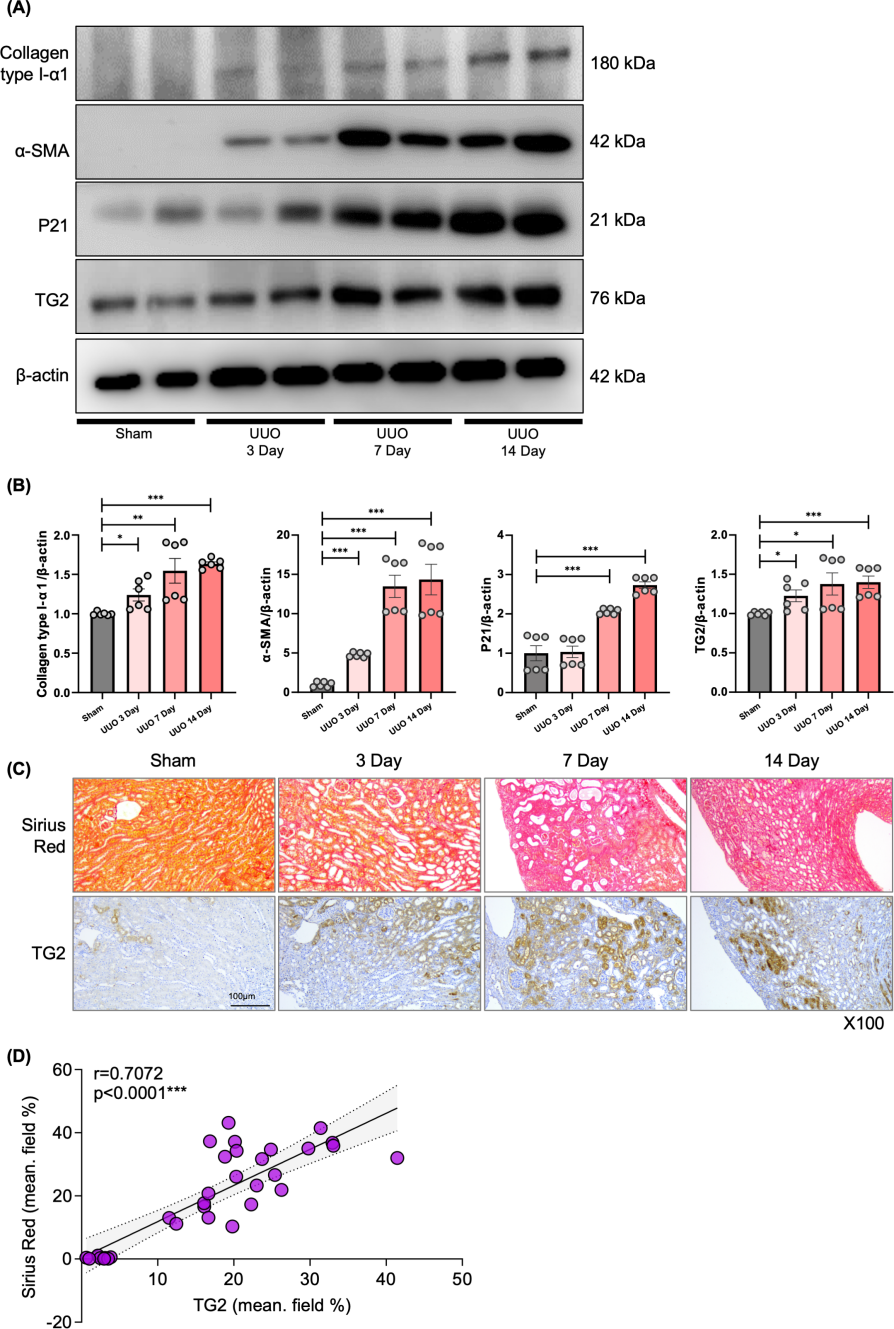
TG2 expression and proteins involved in ECM maintenance, fibroblast activation, and cell cycle correlate with kidney injury severity in UUO kidneys. **(A)** Western blots of collagen type I-α1, α-SMA, P21, TG2, β-actin, and **(B)** densitometric quantification normalized to β-actin. **(C)** Histological staining images of Sirius Red, TG2, and **(D)** Pearson correlation plot. Statistical significance: ^*^ *P* ≤ 0.05, ^**^ *P* ≤ 0.01, ^***^ *P* ≤ 0.001.

We evaluated the expression of TG2 in the UUO mouse model to determine its association with kidney fibrosis. In western blots, the expression of collagen type I-α1, α-SMA, and P21, which are proteins critical in ECM maintenance, fibroblast activation, and cell cycle arrest, respectively, all displayed a positive correlation to kidney injury severity (**Figure 2A**), as well as in the corresponding quantification (**Figure 2B**). In histological staining images, progressive cortical thinning was observed with kidney injury, accompanied by increased TG2 expression (**Figure 2C**) and a corresponding linear increase in fibrosis shown by Sirius Red staining (**Figure 2D**).

### Impact of TG2 inhibition on rTGF-β-induced profibrotic injury

**Figure 3:**
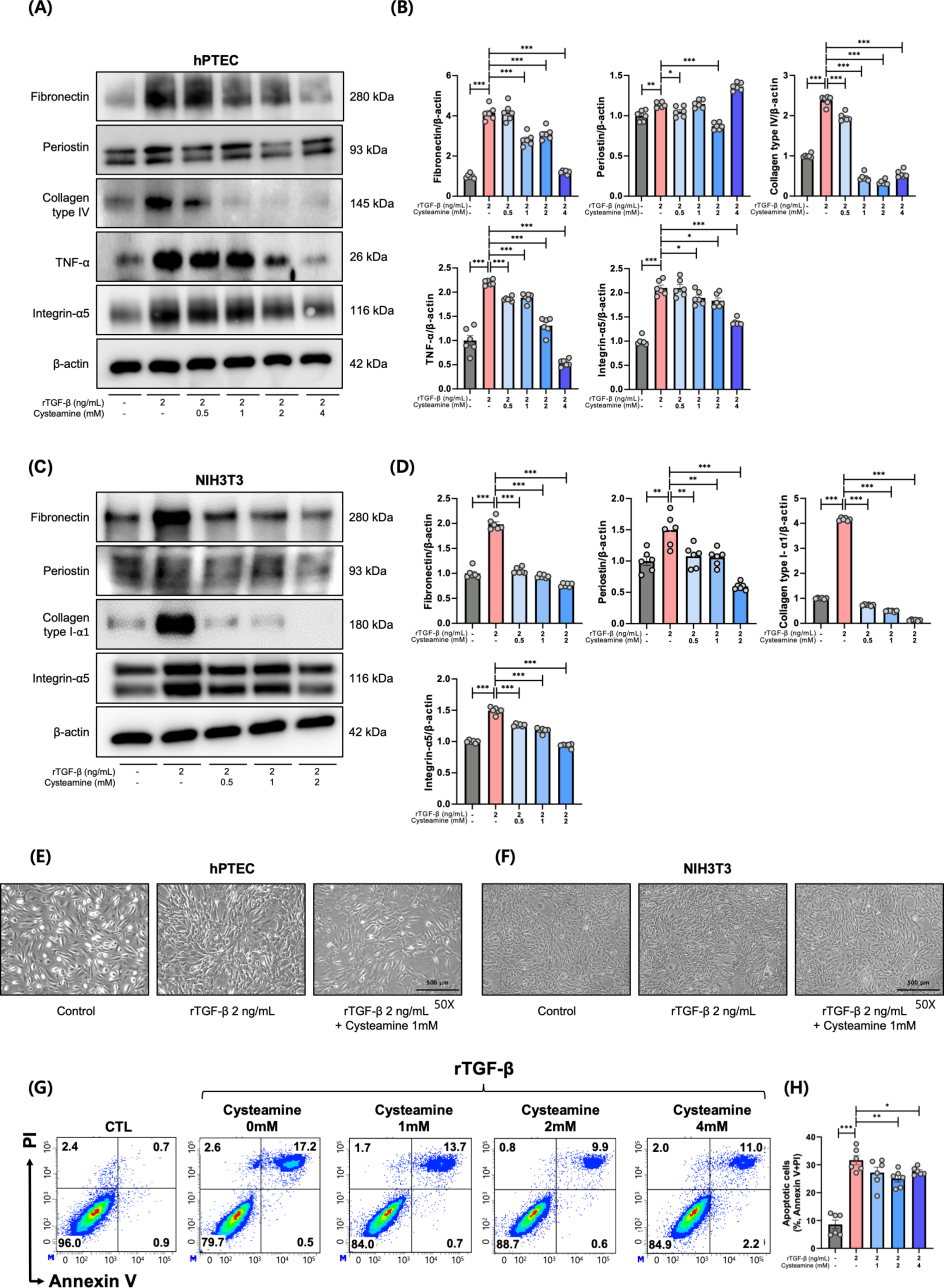
TG2 suppression modulated proteins associated with ECM maintenance, inflammation, apoptosis, and TGF-β signaling in rTGF-β-treated hPTECs and NIH3T3 cells. **(A)** Western blots of fibronectin, periostin, collagen type IV, TNF-α, integrin-α5, and β-actin of hPTECs treated with rTGF-β and cysteamine (48 h), and **(B)** densitometric quantification normalized to β-actin. **(C)** Western blots of fibronectin, periostin, collagen type I-α1, integrin-α5, and β-actin of NIH3T3 cells treated with rTGF-β and cysteamine (24 h), and **(D)** densitometric quantification normalized to β-actin. **(E)** Light microscopic images of hPTECs and **(F)** NIH3T3 cells treated with rTGF-β and cysteamine. **(G)** Annexin V and propidium iodide (PI) staining of human proximal tubular epithelial cells (hPTECs) treated with rTGF-β and cysteamine (48 h), and **(H)** quantification. Statistical significance: ^*^P ≤ 0.05, ^**^P ≤ 0.01, ^***^P ≤ 0.001.

We found a dose-dependent decrease in the heightened expression of ECM-related proteins, including fibronectin, periostin, and collagen type IV, as well as the pro-inflammatory and pro-apoptotic cytokine TNF-α and TGF-β activation-related integrin-α5. Notably, these changes were reversed by TG2 inhibition with cysteamine in hPTECs (**Figures 3A, B**). Similar protein expression patterns were observed in NIH3T3 cells (**Figures 3C, D**). We also observed phenotypic changes, where the naturally cobblestone-like hPTECs transformed into sharper cells with front-back polarity after rTGF-β treatment. However, TG2 inhibition partially restored the cells to their original morphology (**Figures 3E, F**). Apoptosis was markedly increased by rTGF-β treatment compared to control (31.7 ± 1.65% vs 8.6 ± 1.57%, ^***^ *P* < 0.001), and TG2 inhibition with cysteamine significantly attenuated this response at concentrations of 2 mM (25.1 ± 1.25%, ^**^P < 0.01) and 4 mM (27.6 ± 0.56%, ^*^P < 0.05) in a dose-dependent manner, as confirmed by FACS analysis (**Figures 3G, H**).

### Proteomic landscape of differentially expressed proteins in EVs and cluster analysis

**Figure 4:**
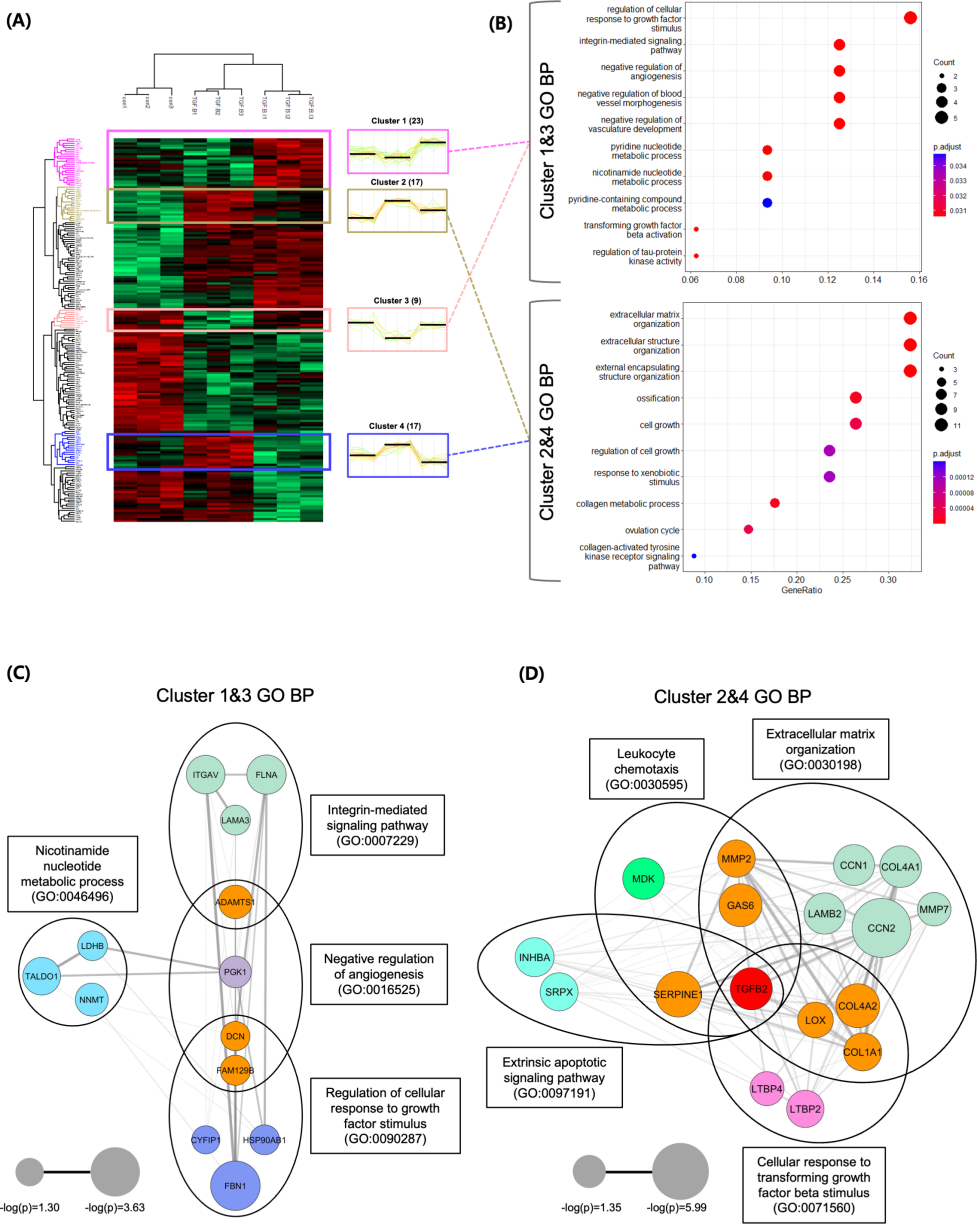
TG2 inhibition influenced protein expression in EVs derived from rTGF-β-treated hPTECs. **(A)** heat map-based clustering, **(B)** GO enrichment analysis, and **(C, D)** STRING network analyses. STRING network analysis was performed using 184 significant DEPs identified via ANOVA and further categorizing the network from 50 hallmark gene sets, modeling well-defined biological processes from the MSigDB Hallmark 2020. Darker lines indicate higher confidence levels of protein pairs, as observed in the STRING analysis.

Next, we evaluated DEPs expressed in hPTEC-derived EVs following treatments with rTGF-β and cysteamine. A total of 537 DEPs were identified (**Supplementary Figure 2**) and were well discriminated according to the treatment conditions in the principal component analysis plot (**Supplementary Figure 3**). We performed one-way ANOVA and found 184 statistically significant DEPs, which were plotted as a heatmap (**Figure 4A**).

The identified DEPs were further clustered based on statistically significant intensity patterns under various treatment conditions. Specifically, clusters 1 and 3 encompassed DEPs that exhibited an initial decrease in intensity following fibrosis induction, followed by an increase in intensity after TG2 inhibition. Conversely, DEPs with contrasting intensity patterns were grouped into clusters 2 and 4. The criteria for distinguishing between clusters 1 and 3 and clusters 2 and 4 were based on baseline expression levels, with clusters 1 and 2 exhibiting significantly lower baseline expression compared to clusters 3 and 4. Finally, we identified 23, 17, 9, and 17 DEPs in clusters 1, 2, 3, and 4, respectively (**Supplementary Table 2**).

Based on the GO enrichment analysis, we identified the biochemical patterns associated with the proteins in each cluster (**Supplementary Table 3**). DEPs in cluster 1 were generally involved in rTGF-β activation and regulation and ECM organization (**Supplementary Figure 4A**). A total of 23 DEPs in cluster 1 included heat shock proteins and decorin, which are involved in cell cycle regulation and apoptosis. Additionally, biglycan, which is presumed to exhibit proinflammatory properties and is often released by macrophages at the onset of kidney injury, is a prominent member of this cluster. The DEPs in cluster 3 were predominantly associated with processes involving smooth muscle cell migration and ECM organization (**Supplementary Figure 4B**). DEPs in cluster 2 were associated with signaling pathways governing cytoskeletal dynamics, notably the Rho GTPase and Wnt signaling pathways (**Supplementary Figure 4C**). DEPs in cluster 4 were primarily related to the Notch signaling pathway and ECM organization (**Supplementary Figure 4D**).

We also combined clusters with similar expression patterns, namely clusters 1, 3, 2, and 4, for an increased gene set size input into the GO enrichment analysis. When placed in the gene ratio hierarchies, the most significant GO Biological Process categories in clusters 1, 3, 2, and 4 were related to cellular responses to growth factor stimuli and ECM organization (**Figure 4B**). Notably, clusters 1 and 3 comprised not only DEPs related to canonically known responses to TGF-β stimulation, but also those that are involved in responses to nucleotide metabolism, which is involved in oxidative stress and inflammation (**Figure 4C**). In clusters 2 and 4, DEPs were not only more interlinked with one another but also had significant relevance to important biological processes such as apoptosis, fibrosis, and inflammation (**Figure 4D**).

### Pairwise analysis of hPTEC EVs

**Figure 5:**
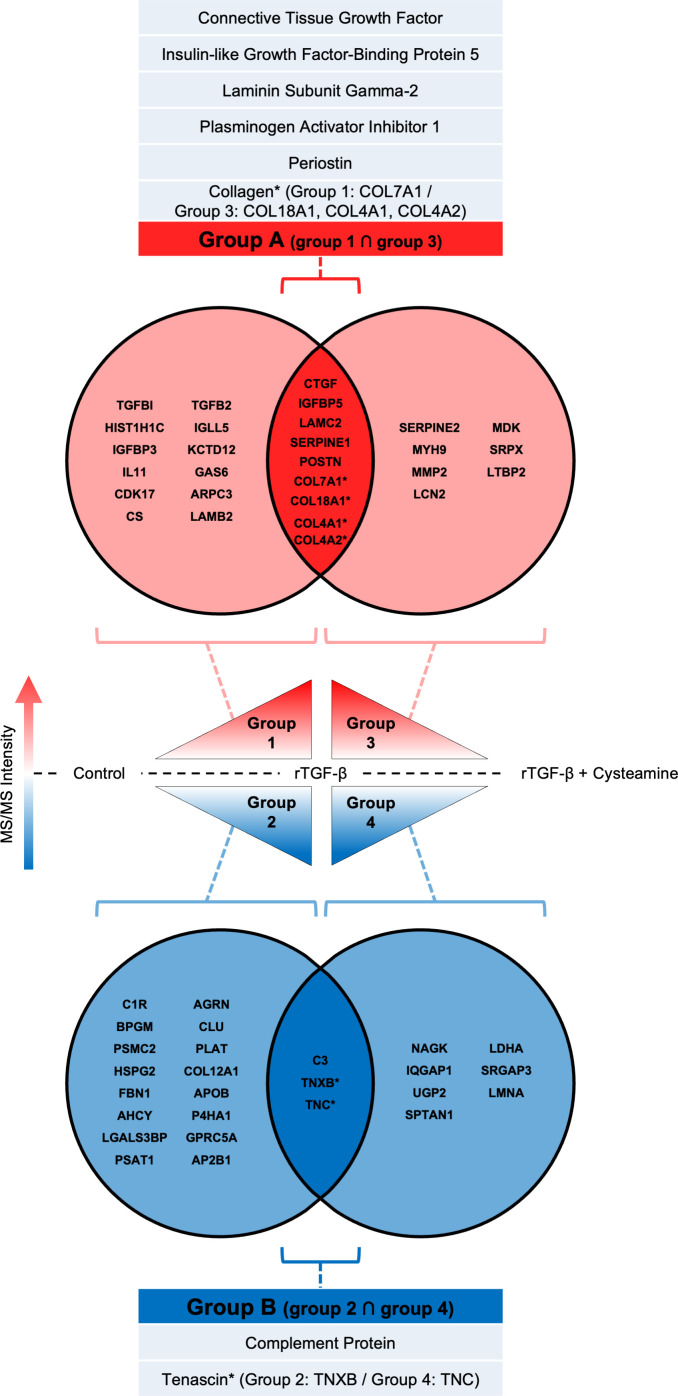
Pairwise comparison of DEPs from rTGF-β-treated hPTEC EVs. Expression changes in Group A and Group B. Group A represented the subset of DEPs shared between Group 1 and Group 3, characterized by an increase following TGF treatment and subsequent decrease after cysteamine administration. Conversely, Group B comprised DEPs shared between Group 2 and Group 4, demonstrating a decrease after TGF treatment and subsequent increase following cysteamine administration.

**Figure 6:**
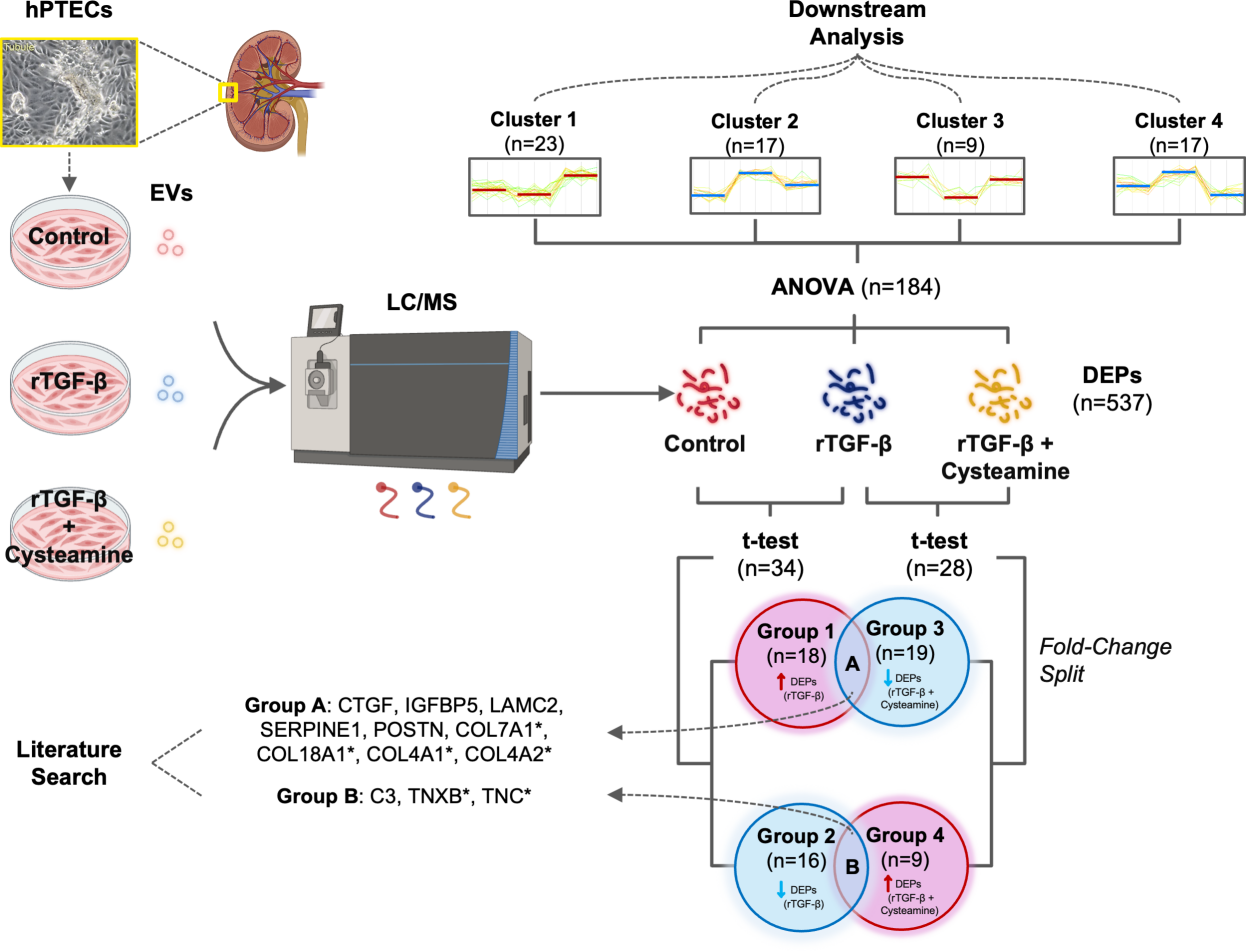
Schematic overview of the proteomic analysis workflow and classification of differentially expressed proteins (DEPs). Extracellular vesicles (EVs) derived from hPTECs under control, rTGF-β, and rTGF-β plus cysteamine conditions were analyzed by LC–MS/MS. A total of 537 DEPs were identified and subjected to one-way ANOVA, yielding 184 significant proteins that were further clustered into four groups based on expression patterns. Pairwise comparisons and fold-change–based classification were then performed to identify DEPs associated with fibrosis induction and reversal. These analyses resulted in the classification of proteins into Group A and Group B intersections, which were subsequently used for downstream biological interpretation and literature-based annotation.

As a part of the *post-hoc* analysis, we conducted a comprehensive examination of the entire proteome via t-tests followed by FDR adjustment. Out of the 537 DEPs, the expression of 34 DEPs exhibited statistical significance upon fibrosis induction, and 28 DEPs were identified upon TG2 suppression after fibrosis induction in hPTECs (**Supplementary Table 4**). DEPs were further categorized based on the fold changes determined by calculating the difference between the mean log_2_-transformed intensity values of each condition. Groups 1 and 2 represent proteins with significantly increased and decreased expression, respectively, in the rTGF-β-treated group compared to the control group. Subsequently, Groups 3 and 4 include proteins with significantly lower and higher expression, respectively, in the rTGF-β and cysteamine-treated group compared to the rTGF-β-treated group. We specifically focused on identifying a set of DEPs at the intersection of groups 1 and 3 (Group A) and another subset at the intersection of groups 2 and 4 (Group B) (**Figure 5**). Group A comprised DEPs shared between groups 1 and 3, consisting of proteins such as plasminogen activator inhibitor 1, laminin subunit gamma 2, insulin-like growth factor-binding protein 5, connective tissue growth factor, and periostin. Concurrently, Group B, representing the intersecting DEPs for Groups 2 and 4, included complement C3- and tenascin-related proteins. An overview of the analytical workflow, from patient-derived hPTECs through EV proteomics to identification of fibrosis-associated proteins, is shown in **Figure 6**.

## Discussion

In this study, we demonstrated that TG2 inhibition significantly alters the EV proteomic landscape under TGF-β–induced fibrotic conditions. EV proteomic analysis identified key differentially expressed proteins associated with ECM organization, inflammatory responses, and cytoskeletal regulation. Notably, several fibrosis-related proteins, including CTGF, IGFBP-5, periostin, and collagen-associated components, exhibited reversible expression patterns following TG2 inhibition, suggesting their potential roles as mediators of fibrosis progression and resolution.

To better interpret these findings, we next examined the role of TG2 in regulating key profibrotic pathways. Enhanced TG2 activity facilitates the crosslinking of latent TGF-β binding protein to fibronectin or other ECM components, thereby playing an indispensable role in TGF-β activation (Kojima et al., 1993). The extracellular release of TG2 plays a crucial role in its functionality during the activation of TGF-β (Shweke et al., 2008). A comprehensive assessment has identified a functional relationship between TG2 and TGF-β involved in the regulatory mechanisms governing angiogenesis and EMT (Wang et al., 2017). In the TGF-β-induced kidney fibrosis model, we found that TG2-mediated kidney fibrosis progresses via an anti-apoptotic mechanism (Moon et al., 2022). In this study, via EV-based proteomics, we provide evidence supporting the roles of TGF-β and TG2 inhibitors in the processes of kidney fibrosis and its restoration. One of the DEPs significantly downregulated upon TG2 inhibition was latent-TGF-β-binding protein 2 (LTBP2). Abundantly expressed in proximal tubular epithelial cells (Ma et al., 2022) and myofibroblasts (Hyytiäinen and Keski-Oja, 2003), LTBP2 is known for its association with TGF-β bioavailability and various renal pathologies (Hyytiäinen and Keski-Oja, 2003; Haase et al., 2014). In addition, we identified a conspicuous association with the biological process related to the regulation of TGF-β activation in cluster 1. The findings suggest that regulation of TGF-β activation may play a more prominent role during the remodeling phase following TG2 inhibition than during the fibrosis induction phase.

The production and deposition of large amounts of ECM components are major cellular events in tubulointerstitial fibrosis (Liu, 2006). The biological pathways of ECM and structural organization were prominent in clusters 3 and 4. Although the response to fibrosis induction was the opposite in clusters 3 and 4, both groups recovered to the control state after TG2 inhibition. The activity of TG2 contributes to the process of fibrosis, especially during the early stages of ECM remodeling (Tatsukawa and Hitomi, 2021). Hence, the recovery from fibrosis could be related to the modulation of the biological process of ECM organization via TG2 inhibition. These findings suggest that TG2-mediated regulation of ECM organization may represent an important mechanism underlying fibrosis reversibility.

While the ECM is vastly dynamic, precise changes in its various components are still under investigation, particularly those involving EVs. Interestingly, co-occurrence of collagen family proteins in groups 1 and 3 and that of tenascin family proteins in groups 2 and 4 was noted. Collagen family proteins contribute to the thickening of the ECM and activation of myofibroblasts, which are the primary drivers of aberrant ECM production during CKD progression (Ruiz-Ortega et al., 2020; Feng et al., 2024). Our results indicated that TG2 inhibition leads to a significant decrease in collagen expression in EVs, potentially reducing its pro-fibrotic effects on the neighboring intercellular environment, along with its inhibition as a collagen crosslinker (Kojima et al., 1993), rendering collagen more susceptible to degradation. Moreover, the collagen alpha-1 (XVIII) chain, known for its endostatin domain that inhibits angiogenesis, was upregulated in another proteomic study of EVs from CKD patients and significantly suppressed upon TG2 inhibition in our study (Jalal et al., 2021). In contrast, the tenascin family proteins, which are key components of the ECM, exhibited regulatory patterns distinct from those of collagen. Notably, tenascin X expression decreased following TGF-β activation, whereas tenascin C expression increased after TG2 inhibition, suggesting differential regulation of ECM components by TG2 inhibition. Previous studies have suggested that tenascin family proteins are involved in modulating immune cell interactions (Yamaguchi et al., 2017; Murdamoothoo et al., 2021). Although these findings raise the possibility that TG2 inhibition may influence immune-related processes through EV-associated proteins, the precise mechanisms in the context of kidney fibrosis remain to be determined.

Interestingly, biological processes related to lamellipodium assembly and Arp2/3 complex-mediated actin nucleation were present in cluster 2, which showed significantly decreased expression after TG2 inhibition. Actin filaments play a crucial role in cellular functions, serving as both a structural scaffold and facilitator of several activities such as cell motility, cell division, intracellular transport of cargo and organelles, and establishment of cell junctions. Specifically, the Arp2/3 complex is required for lamellipodium extension and fibroblast cell migration (Suraneni et al., 2012). Although the relevance of lamellipodium-related processes in kidney fibrosis remains to be fully elucidated (Venkatareddy et al., 2011), our findings suggest that TG2 inhibition may influence cytoskeletal remodeling associated with fibroblast migration. Further studies are required to clarify the role of these pathways in kidney fibrosis.

Considering the serial response to the TGF-β and TG2 inhibition, we examined the specific DEPs associated with the reversibility from the fibrosis process. Connective tissue growth factor (CTGF) is a central mediator of tissue remodeling and fibrosis, and its inhibition has demonstrated the capacity to revert the progression of fibrotic processes (Lipson et al., 2012). CTGF is known to be activated by TGF-β and has been recognized for its involvement in the fibrotic processes across various tissues including the skin, lungs, and vascular system (Igarashi et al., 1993; Sonnylal et al., 2010). Its relevance extends to the context of kidney fibrosis not only in animal models but also in human kidney tissues (Ito et al., 1998; Riser et al., 2000). Higher CTGF expression is significantly associated with a lower estimated glomerular filtration rate and decreased residual kidney function in patients with CKD (Gerritsen et al., 2012). In the present study, CTGF exhibited reversible expression patterns in response to TGF-β stimulation and TG2 inhibition, suggesting its involvement in both fibrosis progression and resolution. These findings further support the potential relevance of CTGF as a therapeutic target in kidney fibrosis.

The growth hormone-insulin-like growth factor axis plays a pivotal role in both the maintenance of normal kidney function and in the pathogenesis and progression of CKD (Oh, 2012). As one of the most abundant and ubiquitous growth factor polypeptides, each class of insulin-like growth factor-binding proteins (IGFBP) is associated with various kidney diseases. Among the IGFBP family, IGFBP-5 is primarily expressed in mesangial cells, and its prominence in glomerular hypertrophy during early diabetes has been reported (Schaeffer et al., 2010). IGFBP-5 is also reported to be significantly expressed in a proteomic study of plasma from CKD patients (Jalal et al., 2021). In our analysis, IGFBP-5 also demonstrated reversible expression patterns following TG2 inhibition, suggesting a potential role in fibrosis-related processes. These findings support the potential utility of IGFBP-5 as a biomarker for kidney fibrosis; however, further validation in clinical samples is required.

Periostin, a matricellular protein primarily expressed in the tubulointerstitium, plays an important role in promoting tissue regeneration, fibrosis, and wound healing by interacting with integrins (Conway et al., 2014). Previous studies have indicated a correlation between urinary periostin levels and tubular damage, suggesting that periostin is a potential indicator of various kidney diseases (Satirapoj et al., 2012, Satirapoj et al., 2015; Wantanasiri et al., 2015; Hwang et al., 2016). We have previously suggested the potential therapeutic efficacy of periostin inhibition in mitigating the progression of CKD based on compelling evidence demonstrating the effective mitigation of renal fibrogenesis through periostin blockade (Hwang et al., 2017). In the present study, periostin exhibited reversible expression patterns in response to TGF-β stimulation and TG2 inhibition, suggesting its involvement in both fibrosis progression and resolution. These findings support the potential relevance of periostin as a therapeutic target in kidney fibrosis.

Plasminogen activator inhibitor 1 (PAI-1) exhibits marked upregulation within the tubulointerstitium, ostensibly responsive to profibrotic cytokines, notably TGF-β (Samarakoon et al., 2012). Mechanistically, PAI-1 contributes to fibrosis by inhibiting plasmin-mediated ECM degradation, thereby promoting matrix accumulation. Consistent with this role, global PAI-1 deficiency has been shown to confer protection against renal fibrosis in experimental models, including UUO, diabetic nephropathy, and chemically induced renal injury (Oda et al., 2001; Nicholas et al., 2005; Eddy and Fogo, 2006). In our analysis, PAI-1 was also associated with fibrosis-related pathways, further supporting its involvement in ECM remodeling processes. Given that renal fibrosis is characterized by excessive deposition of ECM components, such as collagen, fibronectin, and laminins, along with structural alterations including thickening of the tubular and capillary basement membranes (Bülow and Boor, 2019), our findings suggest that PAI-1 may contribute to CKD progression through modulation of ECM turnover and structural remodeling.

The limitations of this study require careful consideration. First, as a non-targeted analysis, this study primarily identified protein changes associated with fibrosis induction and restoration, making it difficult to determine the precise mechanistic roles of individual proteins. Second, although *in vivo* experiments were performed to evaluate the role of TG2 in kidney fibrosis, EV-based analyses were not conducted in the UUO model. Finally, several fibrosis-associated proteins identified in this study were not validated in patient-derived CKD samples. Future studies are warranted to validate the identified EV-associated proteins in human CKD cohorts and to further elucidate their functional roles in fibrosis-related processes. In particular, investigation of TG2-mediated EV signaling pathways may provide novel therapeutic opportunities. In addition, EV-based biomarkers have potential for clinical application in the diagnosis and monitoring of kidney fibrosis, although validation in large-scale patient populations will be required.

## Conclusion

In conclusion, inhibition of TG2 significantly mitigated fibrosis induced by TGF-β. In the trajectory of fibrosis induction and subsequent restoration, the identification of factors exhibiting reversible characteristics is pivotal for identifying potential therapeutic targets. Collectively, our findings highlight TG2 as a key regulator of EV-mediated fibrotic signaling. Furthermore, EV-associated proteins exhibiting reversible expression patterns, including CTGF, IGFBP-5, periostin, PAI, laminin, and collagen-related components, may serve as promising therapeutic targets in kidney fibrosis.

## ARRIVE guidelines statement

All animal experiments were carried out in accordance with relevant guidelines and regulations. Experimental protocols were approved by the Seoul National University Hospital Institutional Animal Care and Use Committee (permit number: 20-0009-S1A0). All procedures followed the ARRIVE (Animal Research: Reporting of *In Vivo* Experiments) guidelines.

## Data Availability

The data presented in the study are deposited in the ProteomeXchange Consortium repository via the PRIDE partner repository, accession number PXD055260.
