# Supplementary material for: A comprehensive approach to elucidating the pathophysiology of kidney fibrosis based on extracellular vesicle proteomics
Source: Front Physiol. 2026 May 29;17:1786999. doi: 10.3389/fphys.2026.1786999 (PMC13259983; doi:10.3389/fphys.2026.1786999)
Supplement: Supplementary file 1 [file DataSheet1.docx]

**Supplemental Materials**

**Supplemental Table 1. The list of antibodies used in western blot analysis**

| **Antibodies** | **Vendor** | **Catalog Number** | **Dilution** |
| --- | --- | --- | --- |
| anti-β-actin (human, mouse) | Sigma-Aldrich, St. Louis, MO, USA | A1978 | 1/10,000 |
| anti-fibronectin (human, mouse) | Abcam, Cambridge, MA, USA | ab2413 | 1/500 |
| anti-collagen type I 𝛂1 (human, mouse) | GeneTex, Irvine, CA, USA | GTX112731 | 1/1,000 |
| anti-collagen type I 𝛂1 (mouse) | Santa Cruz Biotechnology, Dallas, TX, USA | sc293182 | 1/150 |
| anti-collagen type IV (human, mouse) | Abcam, Cambridge, MA, USA | ab6586 | 1/500 |
| anti-periostin (human, mouse) | Abcam, Cambridge, MA, USA | ab152099 | 1/1,000 |
| anti-𝛂-smooth muscle actin (mouse) | Abcam, Cambridge, MA, USA | ab32575 | 1/1,000 |
| anti-P21 (mouse) | Santa Cruz Biotechnology, Dallas, TX, USA | sc6246 | 1/1,000 |
| anti-CD63 (human) | Invitrogen, Carlsbad, CA, USA | PA5-92370 | 1/1,000 |
| anti-CD81 (human) | Santa Cruz Biotechnology, Dallas, TX, USA | sc166029 | 1/250 |
| anti-calnexin (human) | Proteintech, Chicago, IL, USA | 66903-1-ig | 1/10,000 |
| anti-tumor necrosis factor 𝛂 (human, mouse) | Abcam, Cambridge, MA, USA | ab66579 | 1/1,000 |
| anti-integrin 𝛂5 (human, mouse) | Abcam, Cambridge, MA, USA | ab179475 | 1/1,000 |
| anti-transglutaminase 2 (human, mouse) | Abcam, Cambridge, MA, USA | ab421 | 1/500 |

**Supplemental Table 2. Differentially expressed proteins in each cluster**

|  | **Differentially expressed proteins** |
| --- | --- |
| **Cluster 1** | L-lactate dehydrogenase A chain, N-acetyl-D-glucosamine kinase, Filamin-A, Integrin alpha-V;Integrin alpha-V heavy chain;Integrin alpha-V light chain, Transaldolase, Alpha-actinin-1, Spectrin alpha chain, non-erythrocytic 1, UTP--glucose-1-phosphate uridylyltransferase, Nicotinamide N-methyltransferase, Proteasome subunit alpha type-7, Phosphoglycerate kinase 1, Niban-like protein 1, Vimentin, Heat shock protein HSP 90-beta, Laminin subunit alpha-3, Biglycan, L-lactate dehydrogenase B chain;L-lactate dehydrogenase, Adenosine kinase, Decorin, Inactive tyrosine-protein kinase 7, 60S ribosomal protein L18, Ferritin heavy chain;Ferritin heavy chain, N-terminally processed;Ferritin, Ras-related protein Rab-6B |
| **Cluster 2** | Connective tissue growth factor, Insulin-like growth factor-binding protein 5, Laminin subunit gamma-2, Plasminogen activator inhibitor 1, Growth arrest-specific protein 6, Insulin-like growth factor-binding protein 3, Transforming growth factor beta-2;Latency-associated peptide, Histone H1.2, Laminin subunit beta-2, Adenylyl cyclase-associated protein 1, Inhibin beta A chain, Elongation factor 1-delta, Collagen alpha-1(I) chain, 72 kDa type IV collagenase;PEX, 40S ribosomal protein S18, Endothelial lipase, Matrilysin |
| **Cluster 3** | Fibrillin-1, Clusterin;Clusterin beta chain;Clusterin alpha chain;Clusterin, A disintegrin and metalloproteinase with thrombospondin motifs 1, 26S proteasome non-ATPase regulatory subunit 3, AP-2 complex subunit beta, SLIT-ROBO Rho GTPase-activating protein 3, Fibrous sheath-interacting protein 2, Keratin, type I cytoskeletal 18, Cytoplasmic FMR1-interacting protein 1 |
| **Cluster 4** | Collagen alpha-2(IV) chain;Canstatin, Glia-derived nexin, BTB/POZ domain-containing protein KCTD12, Collagen alpha-1(IV) chain;Arresten, Midkine, Protein CYR61, Insulin-like growth factor-binding protein 7, Sushi repeat-containing protein SRPX, Latent-transforming growth factor beta-binding protein 2, Interleukin-11, Procollagen C-endopeptidase enhancer 1, Protein-lysine 6-oxidase, Calsyntenin-1;Soluble Alc-alpha;CTF1-alpha, Latent-transforming growth factor beta-binding protein 4, Myosin light polypeptide 6, Oncoprotein-induced transcript 3 protein, Peptidyl-glycine alpha-amidating monooxygenase;Peptidylglycine alpha-hydroxylating monooxygenase;Peptidyl-alpha-hydroxyglycine alpha-amidating lyase |

**Supplemental Table 3. List of differentially expressed genes and related gene ontology according to each cluster**

|  | **Differentially expressed gene** | **Gene ontology** |
| --- | --- | --- |
| **Cluster 1** | LDHA, NAGK, FLNA, ITGAV, TALDO1, ACTN1, SPTAN1, UGP2, NNMT, PSMA7, PGK1, FAM129B, VIM, HSP90AB1, LAMA3, BGN, LDHB, ADK, DCN, PTK7, RPL18, FTH1, RAB6B | Regulation of transforming growth factor beta activation (GO:1901388)  Pyruvate metabolism process (GO:0006090)  Protein localization to golgi apparatus (GO:0034067)  Glycolytic process (GO:0006096)  Endodermal cell differentiation (GO:0035987)  Intra-golgi vesicle-mediated transport (GO:0006891)  Negative regulation of protein metabolic process (GO:0051248)  Platelet aggregation (GO:0070527)  Endoderm formation (GO:0001706)  Carbohydrate catabolic process (GO:0016052) |
| **Cluster 2** | CTGF, IGFBP5, AMC2, SERPINE1, GAS6, IGFBP3, TGFB2, HIST1H1C, LAMB2, CAP1, INHBA, EEF1D, COL1A1, MMP2, RPS18, LIPG, MMP7 | Lamellipodium assembly (GO:0030032)  Neuron projection guidance (GO:0097485)  Axon guidance (GO:0007411)  Protein import (GO:0017038)  Negative regulation of neuron migration (GO:2001223)  Maintenance of protein location in extracellular region (GO:0071694)  Positive regulation of Arp2/3 compex-mediated actin nucleation (GO:2000601)  Axonogenesis (GO:0007409)  Response to misfolded protein (GO:0051788)  Positive regulation of protein metabolic process (GO:0051247) |
| **Cluster 3** | FBN1, CLU, ADAMTS1, PSMD3, AP2B1, SRGAP3, FSIP2, KRT18, CYFIP1 | Negative regulation of smooth muscle cell migration (GO:0014912)  Extracellular matrix organization (GO:0030198)  Regulation of smooth muscle cell migration (GO:0014910)  Odontogenesis (GO:0042476)  Positive regulation of multicellular organismal process (GO:0051240)  Regulation of cell migration (GO:0030334)  Positive regulation of cell migration (GO:0030335)  Positive regulation of cell differentiation (GO:0045597)  Extracellular structure organization (GO:0043062)  Regulation of insulin-like growth factor receptor signaling pathway (GO:0043567) |
| **Cluster 4** | COL4A2, SERPINE2, KCTD12, COL4A1, MDK, CYR61, IGFBP7, SRPX, LTBP2, IL11, PCOLCE, LOX, CLSTN1, LTBP4, MYL6, OIT3, PAM | Positive regulation of glial cell differentiation (GO:0045687)  Extracellular matrix organization (GO:0030198)  Negative regulation of epithelial cell apoptosis process (GO:1904036)  Blood vessel morphogenesis (GO:0048514)  Extracellular structure organization (GO:0043062)  External encapsulating structure organization (GO:0045229)  Regulation of hepatocyte proliferation (GO:2000345)  Negative regulation of cardiac muscle cell apoptotic process (GO:0010667)  Regulation of neutrophil extravasation (GO:2000389)  Leukocyte chemotaxis involved in inflammatory response (GO:0002232) |

**Supplemental Table 4. Significant proteins in pairwise comparison of EV DEPs after treatment with rTGF-β and cysteamine**

| **Group** | **Gene names** | **Protein names** | **Fold-change** |
| --- | --- | --- | --- |
| Group 1 | TGFBI | Transforming growth factor-beta-induced protein ig-h3 | 2.83 |
|  | **CTGF** | **Connective tissue growth factor** | **3.35** |
|  | COL7A1 | Collagen alpha-1(VII) chain | 1.46 |
|  | IGFBP3 | Insulin-like growth factor-binding protein 3 | 3.67 |
|  | **IGFBP5** | **Insulin-like growth factor-binding protein 5** | **2.91** |
|  | IL11 | Interleukin-11 | 3.83 |
|  | CDK17 | Cyclin-dependent kinase 17 | 3.74 |
|  | CS | Citrate synthase;Citrate synthase, mitochondrial | 2.68 |
|  | **LAMC2** | **Laminin subunit gamma-2** | **2.12** |
|  | TGFB2 | Transforming growth factor beta-2;Latency-associated peptide | 1.36 |
|  | IGLL5;IGLV2-11;IGLV2-8 | Ig lambda chain V-II region WIN;Ig lambda chain V-II region MGC;Ig lambda chain V-II region TRO;Ig lambda chain V-I region HA;Immunoglobulin lambda-like polypeptide 5 | 1.94 |
|  | KCTD12 | BTB/POZ domain-containing protein KCTD12 | 1.78 |
|  | **SERPINE1** | **Plasminogen activator inhibitor 1** | **1.96** |
|  | GAS6 | Growth arrest-specific protein 6 | 1.55 |
|  | ARPC3 | Actin-related protein 2/3 complex subunit 3 | 2.42 |
|  | LAMB2 | Laminin subunit beta-2 | 1.70 |
|  | HIST1H1C;HIST1H1E;HIST1H1D | Histone H1.2;Histone H1.4;Histone H1.3 | 1.23 |
|  | **POSTN** | **Periostin** | **1.32** |
| Group 2 | **C3** | **Complement (C) C3; C3 β-chain; C3 α-chain; C3a anaphylatoxin; Acylation stimulating protein; C3b α-chain; C3c α-chain fragment1; C3dg fragment; C3g fragment; C3d fragment; C3f fragment; C3c α-chain fragment2** | **-1.11** |
|  | C1R | Complement C1r subcomponent;Complement C1r subcomponent heavy chain;Complement C1r subcomponent light chain | -0.87 |
|  | BPGM | Bisphosphoglycerate mutase | -2.12 |
|  | PSMC2 | 26S protease regulatory subunit 7 | -1.36 |
|  | HSPG2 | Basement membrane-specific heparan sulfate proteoglycan core protein;Endorepellin;LG3 peptide | -1.42 |
|  | FBN1 | Fibrillin-1 | -0.58 |
|  | AHCY | Adenosylhomocysteinase | -0.72 |
|  | LGALS3BP | Galectin-3-binding protein | -0.77 |
|  | PSAT1 | Phosphoserine aminotransferase | -2.40 |
|  | HIST1H4A | Histone H4 | -0.59 |
|  | AGRN | Agrin;Agrin N-terminal 110 kDa subunit;Agrin C-terminal 110 kDa subunit;Agrin C-terminal 90 kDa fragment;Agrin C-terminal 22 kDa fragment | -1.07 |
|  | CLU | Clusterin;Clusterin beta chain;Clusterin alpha chain;Clusterin | -0.70 |
|  | **TNXB** | **Tenascin-X** | **-0.62** |
|  | PLAT | Tissue-type plasminogen activator;Tissue-type plasminogen activator chain A;Tissue-type plasminogen activator chain B | -1.29 |
|  | COL12A1 | Collagen alpha-1(XII) chain | -0.45 |
|  | APOB | Apolipoprotein B-100;Apolipoprotein B-48 | -0.71 |

**Continued**

| **Group** | **Gene names** | **Protein names** | **Fold-change** |
| --- | --- | --- | --- |
| Group 3 | **SERPINE1** | **Plasminogen activator inhibitor 1** | **1.27** |
|  | SERPINE2 | Glia-derived nexin | 1.17 |
|  | MYH9 | Myosin-9 | 0.62 |
|  | COL18A1 | Collagen alpha-1(XVIII) chain;Endostatin | 0.91 |
|  | MMP2 | 72 kDa type IV collagenase;PEX | 1.72 |
|  | **LAMC2** | **Laminin subunit gamma-2** | **0.76** |
|  | COL4A1 | Collagen alpha-1(IV) chain;Arresten | 1.16 |
|  | **IGFBP5** | **Insulin-like growth factor-binding protein 5** | **1.91** |
|  | **CTGF** | **Connective tissue growth factor** | **1.23** |
|  | MDK | Midkine | 2.35 |
|  | SRPX | Sushi repeat-containing protein SRPX | 0.83 |
|  | **POSTN** | **Periostin** | **1.84** |
|  | COL4A2 | Collagen alpha-2(IV) chain;Canstatin | 1.25 |
|  | LTBP2 | Latent-transforming growth factor beta-binding protein 2 | 1.67 |
|  | LCN2 | Neutrophil gelatinase-associated lipocalin | 3.26 |
|  | **POSTN** | **Periostin** | **4.76** |
|  | **SERPINE1** | **Plasminogen activator inhibitor 1** | **1.27** |
|  | SERPINE2 | Glia-derived nexin | 1.17 |
|  | MYH9 | Myosin-9 | 0.62 |
| Group 4 | NAGK | N-acetyl-D-glucosamine kinase | -3.56 |
|  | **TNC** | **Tenascin** | **-1.20** |
|  | IQGAP1 | Ras GTPase-activating-like protein IQGAP1 | -1.46 |
|  | **C3** | **Complement (C) C3; C3 β-chain; C3 α-chain; C3a anaphylatoxin; Acylation stimulating protein; C3b α-chain; C3c α-chain fragment1; C3dg fragment; C3g fragment; C3d fragment; C3f fragment; C3c α-chain fragment2** | **-0.37** |
|  | UGP2 | UTP--glucose-1-phosphate uridylyltransferase | -0.96 |
|  | LDHA | L-lactate dehydrogenase A chain | -0.59 |
|  | SRGAP3 | SLIT-ROBO Rho GTPase-activating protein 3 | -1.30 |
|  | LMNA | Prelamin-A/C;Lamin-A/C | -0.63 |
|  | SPTAN1 | Spectrin alpha chain, non-erythrocytic 1 | -1.06 |

The 34 DEPs identified during fibrosis induction were further divided into groups 1 (positive fold change) and 2 (negative fold change) based on the intensity ratio of rTGF-β group / control group. Likewise, the 28 DEPs detected during TG2 inhibition after fibrosis induction were subdivided into groups 3 (positive fold change) and 4 (negative fold change) based on the intensity ratio of rTGF-β + cysteamine group / rTGF-β group.

**(A)**

| **CD63**  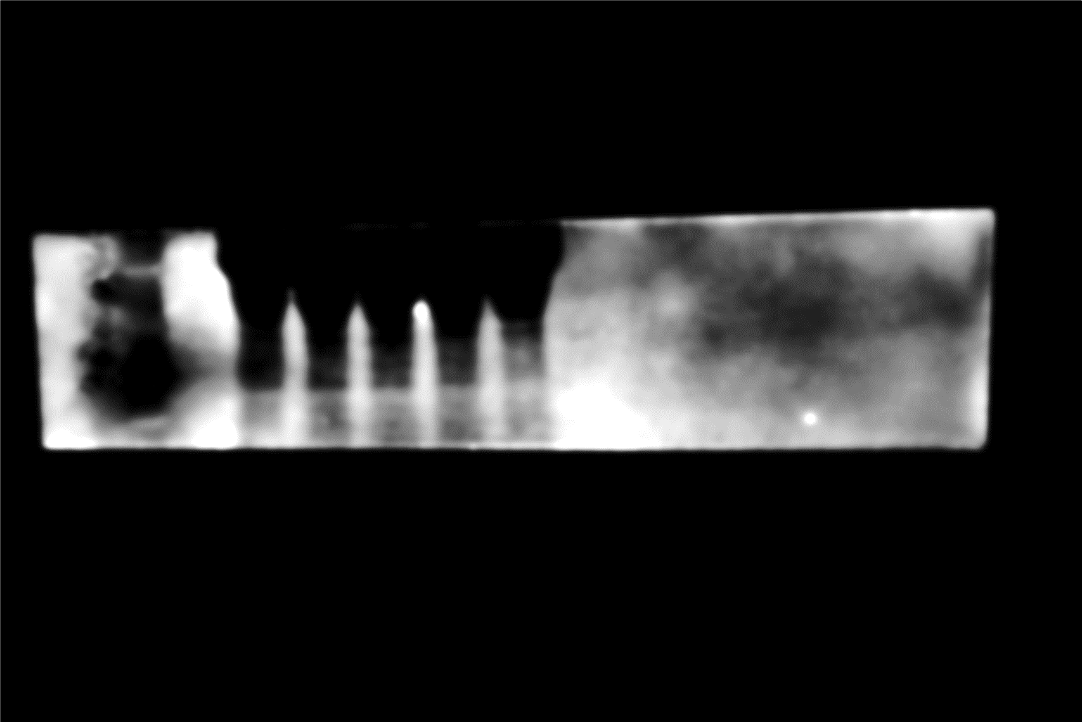 | **CD81**  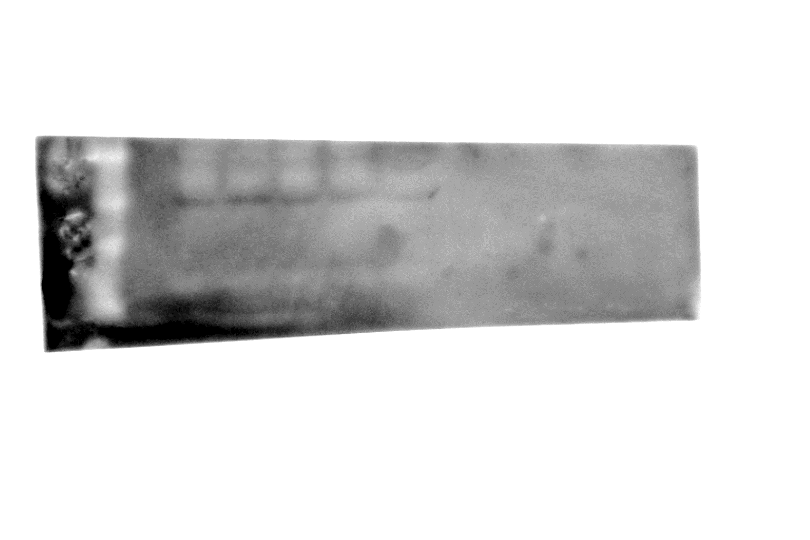 |
| --- | --- |

**(B)**

| **Calnexin**  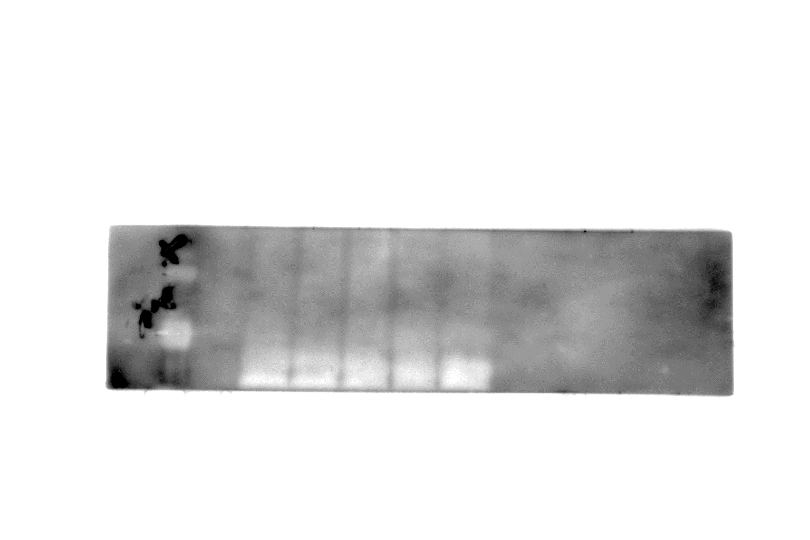 |  |
| --- | --- |

**(C)**

| **Collagen Type I-α1**  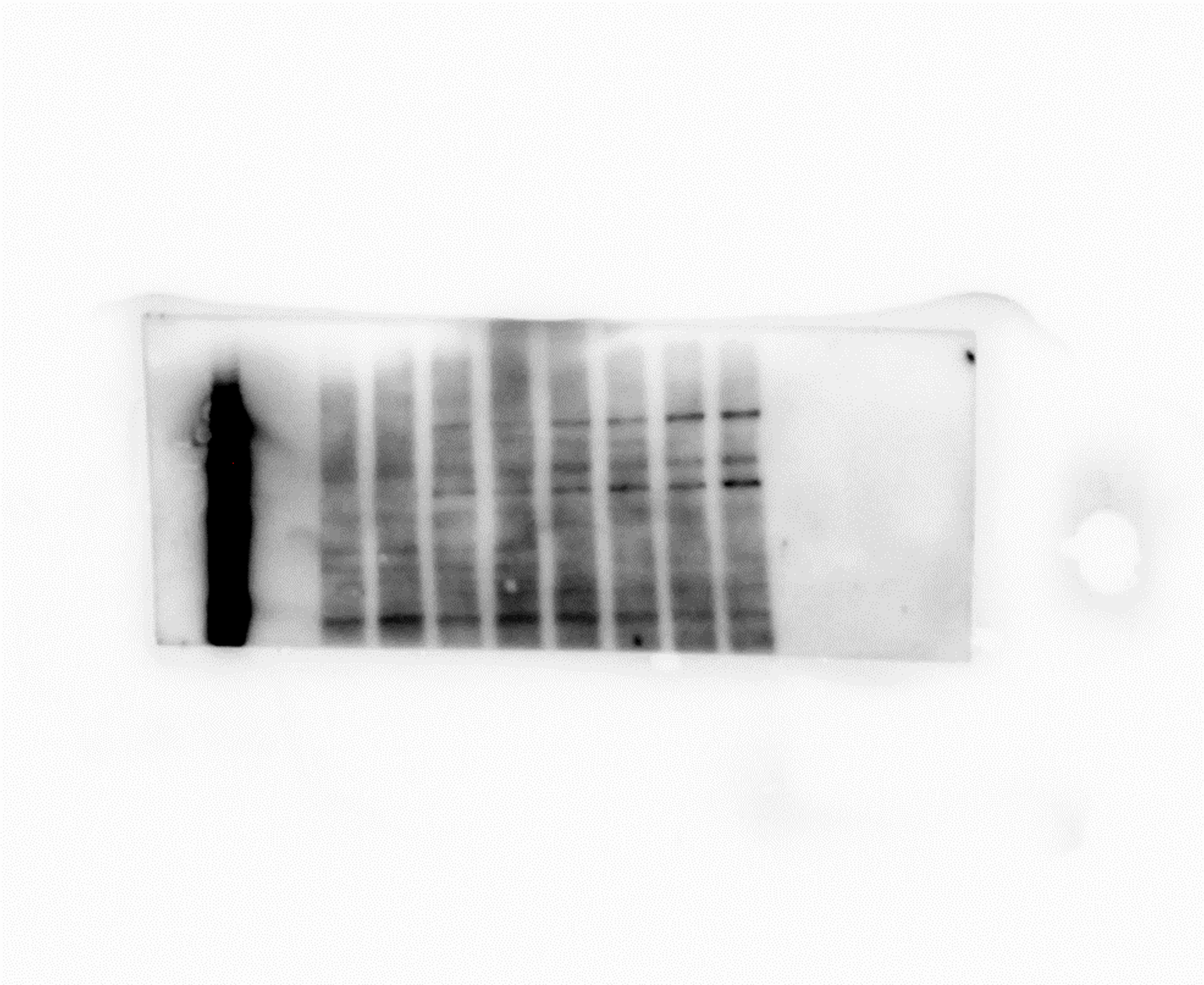 | **α-SMA**  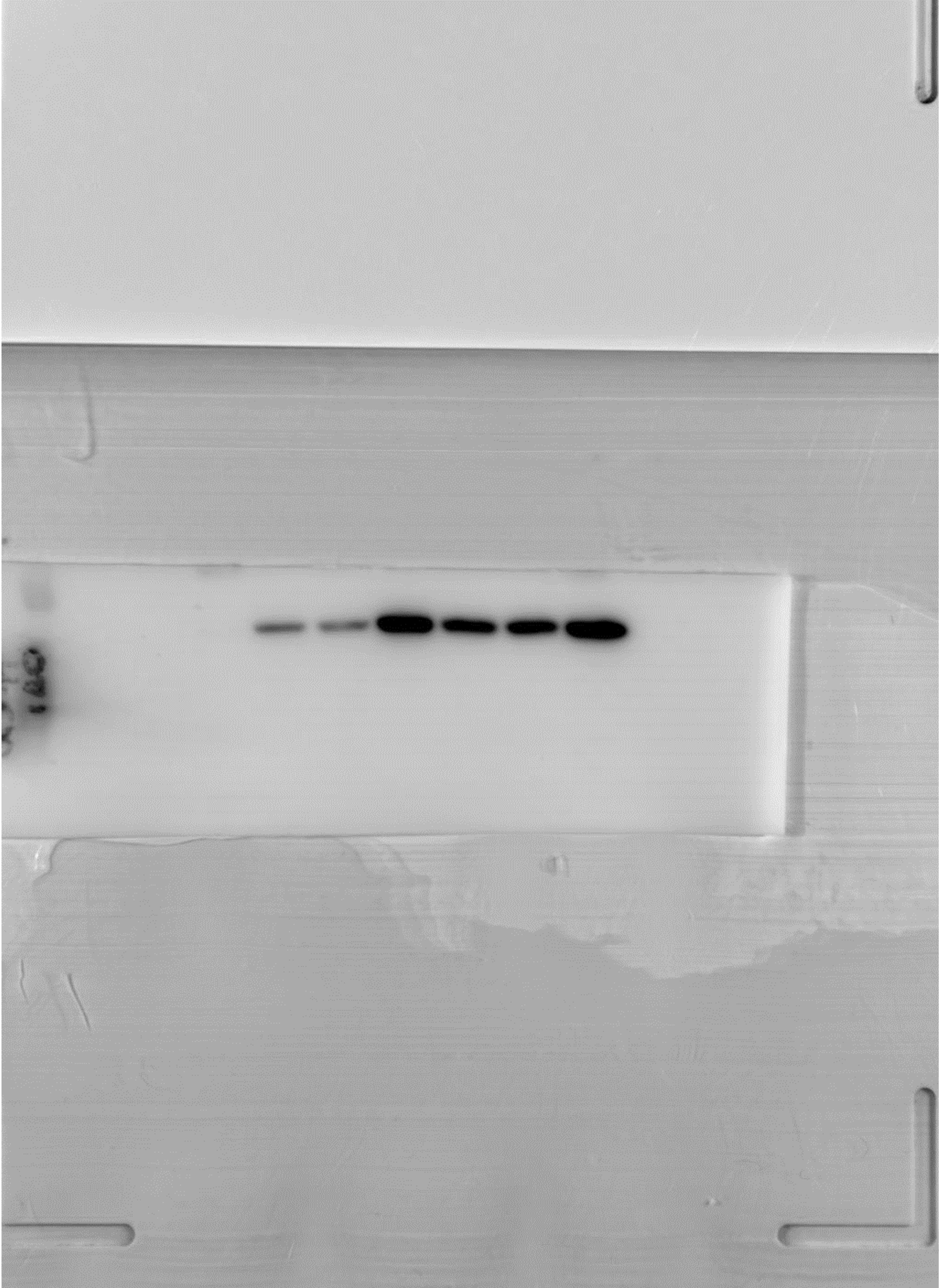 |
| --- | --- |

**(D)**

| **P21**  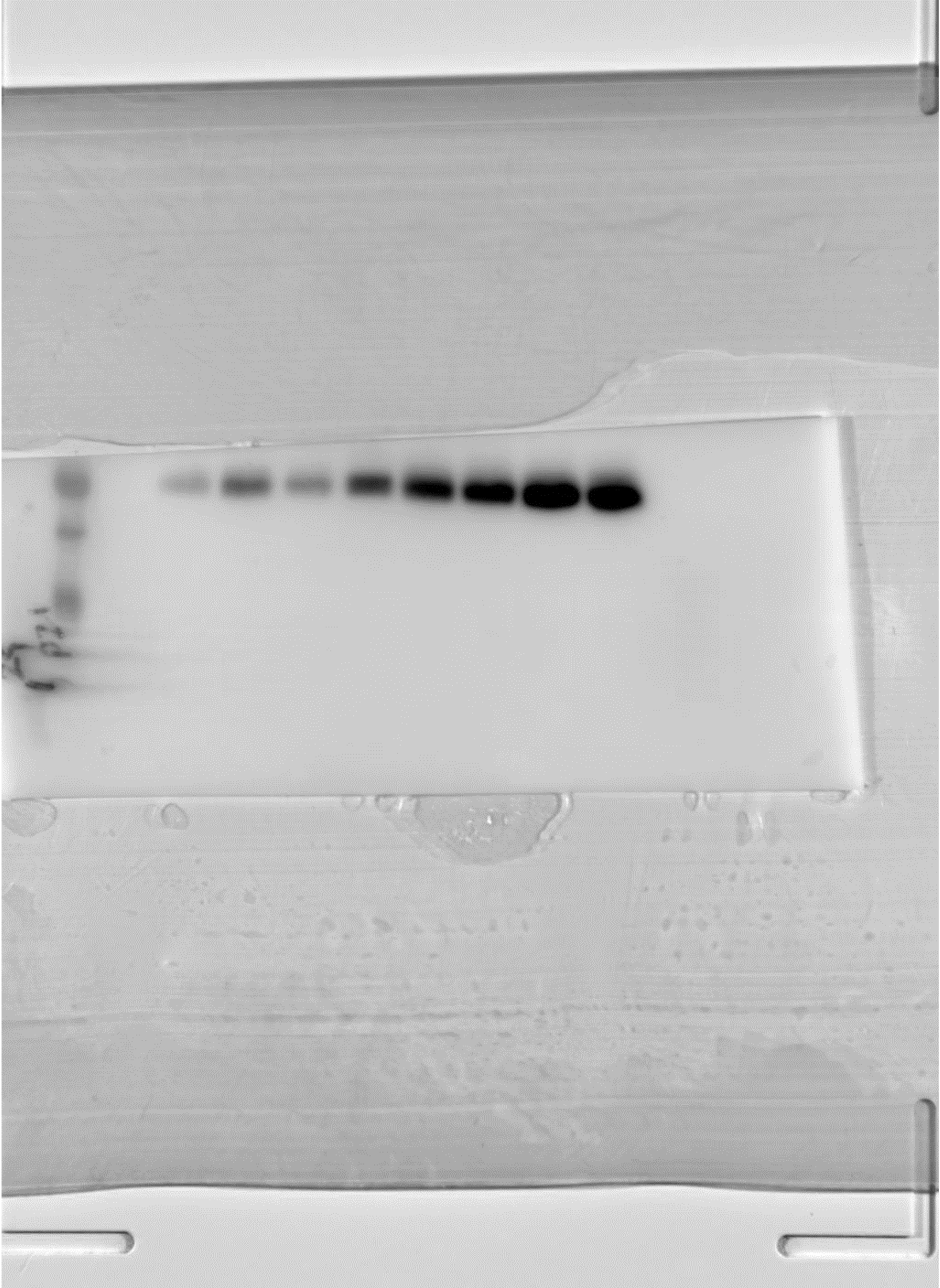 | **TG2**  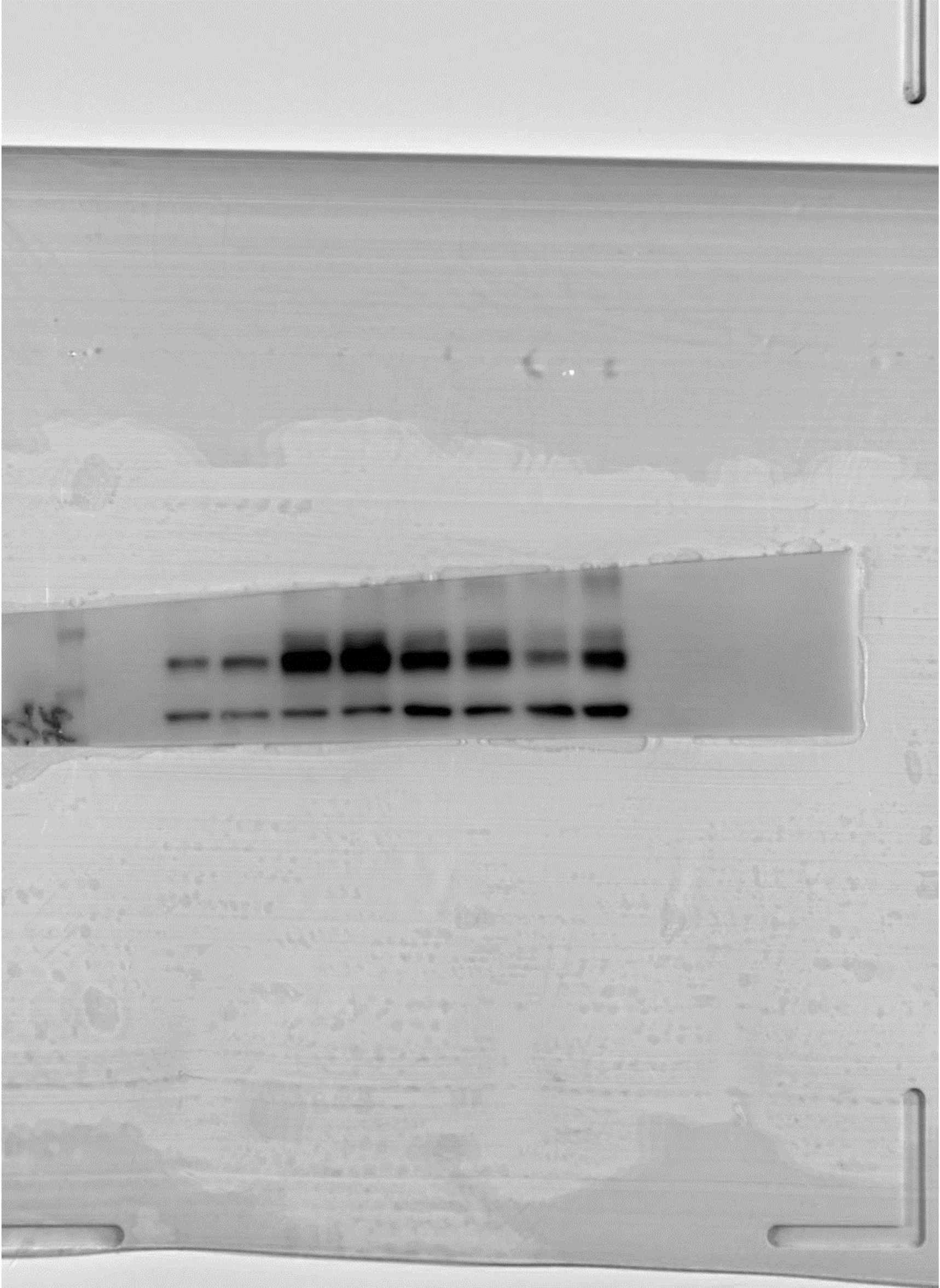 |
| --- | --- |

**(E)**

| **β-Actin**  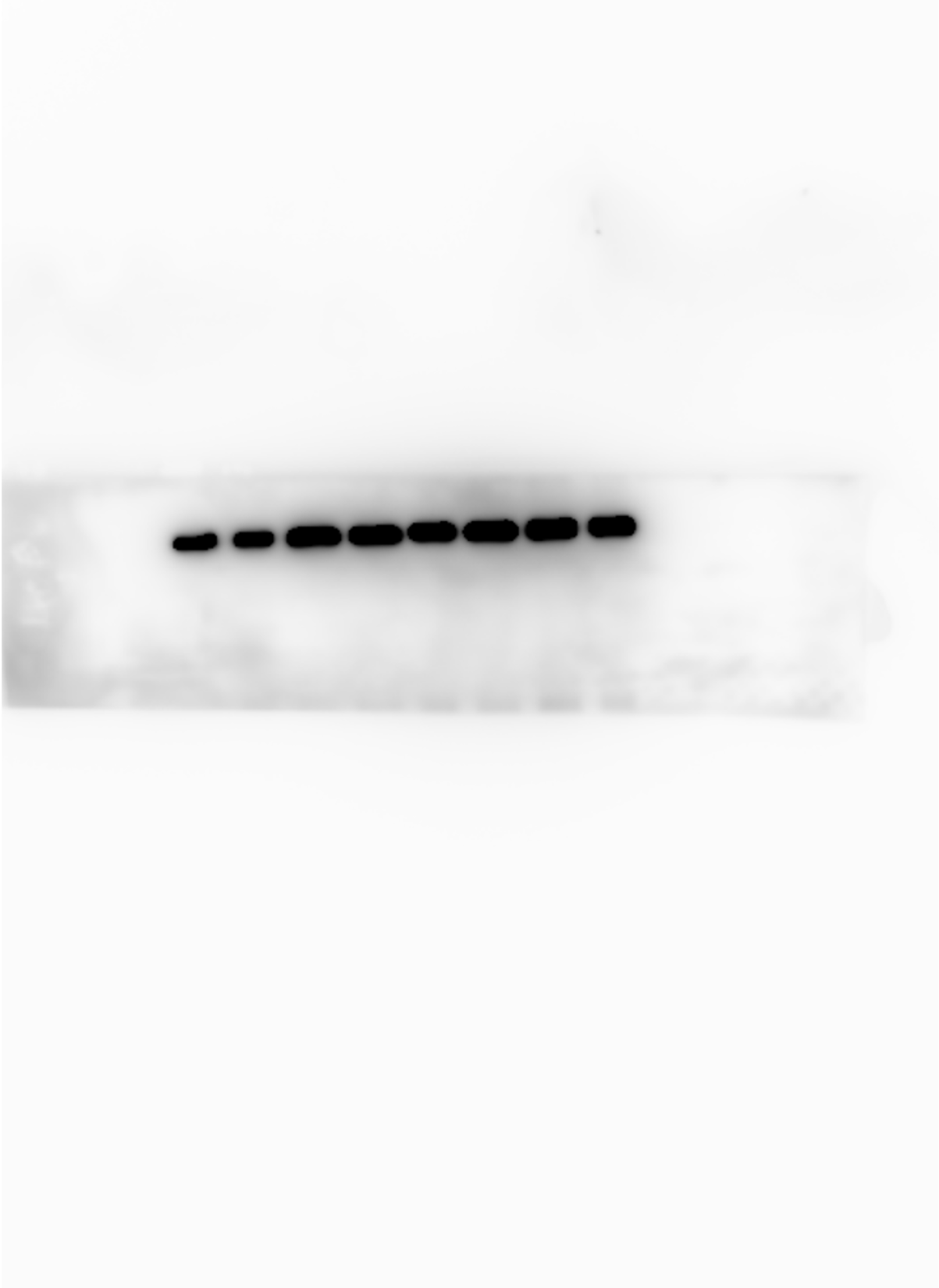 |  |
| --- | --- |

**(F)**

| **Fibronectin**  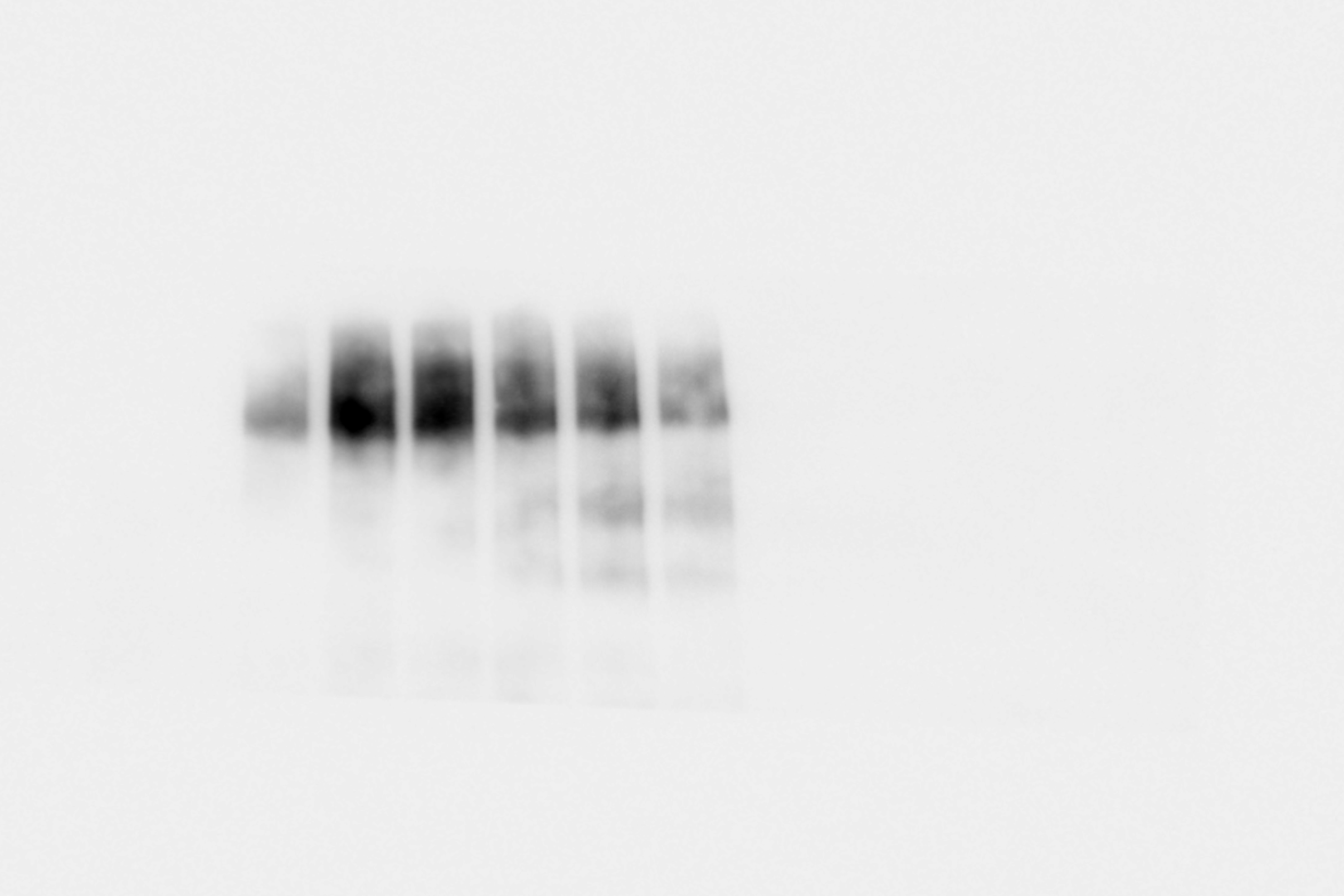 | **Periostin**  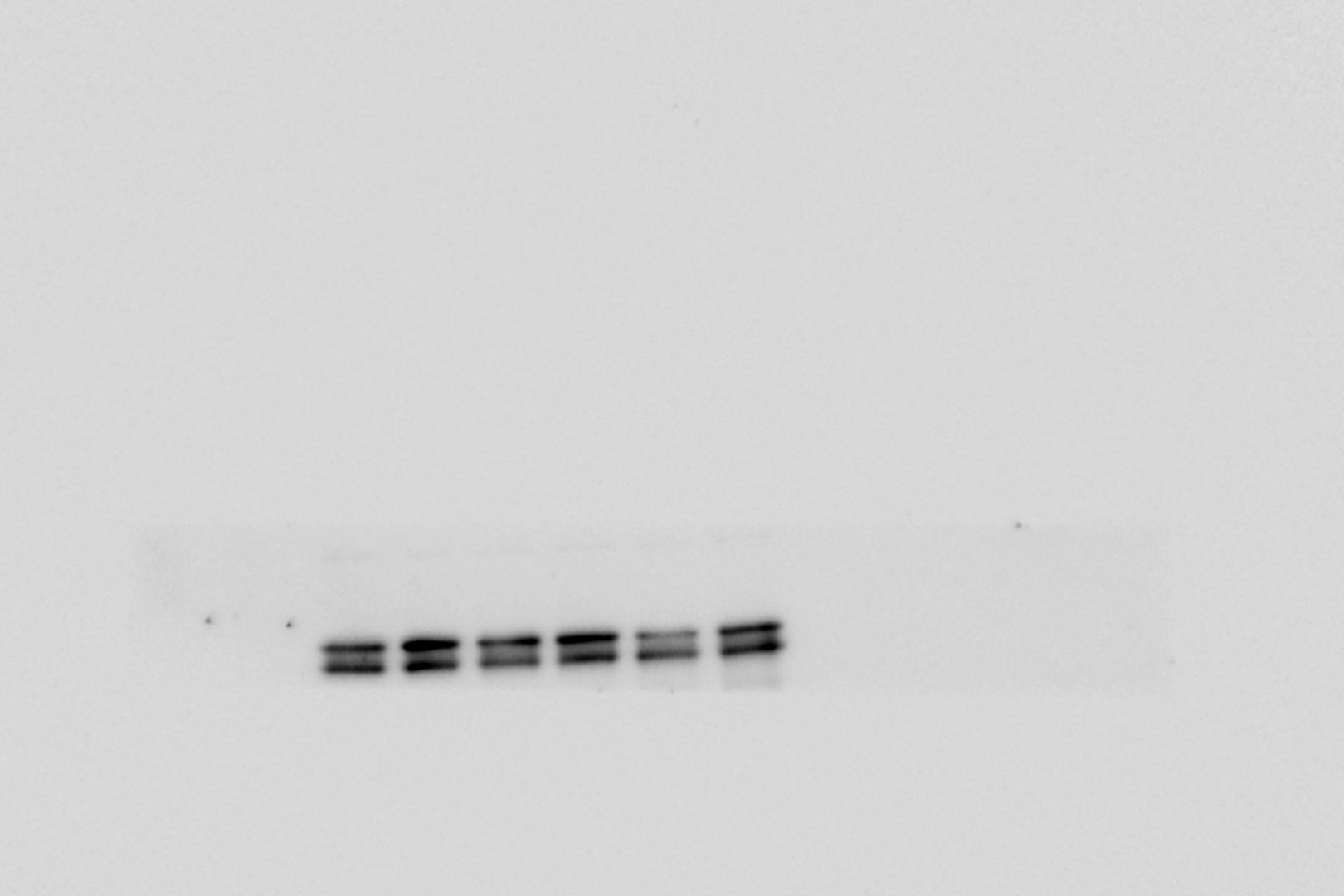 |
| --- | --- |

**(G)**

| **Collagen Type IV**  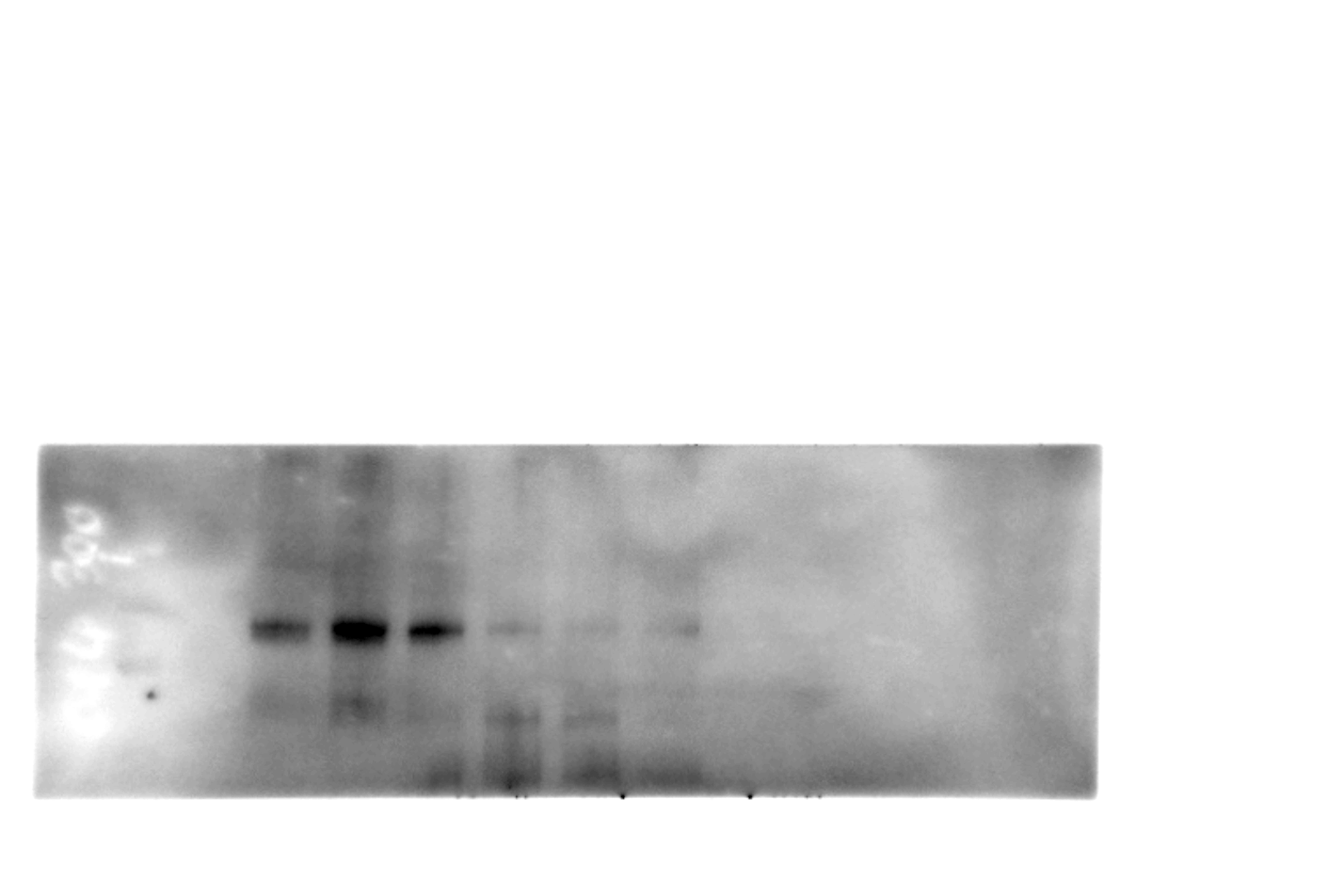 | **TNF-α**  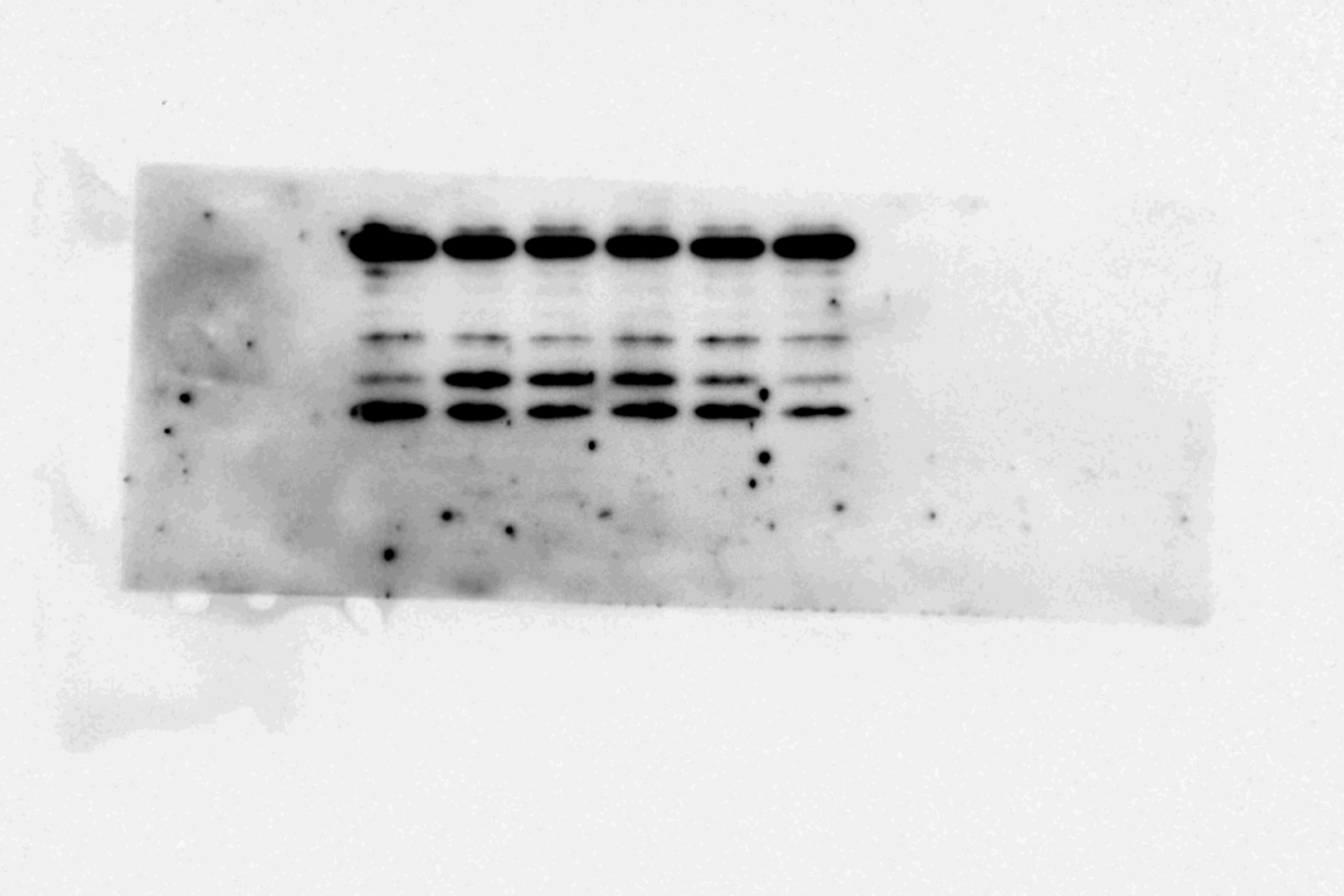 |
| --- | --- |

**(H)**

| **Integrin-α5**  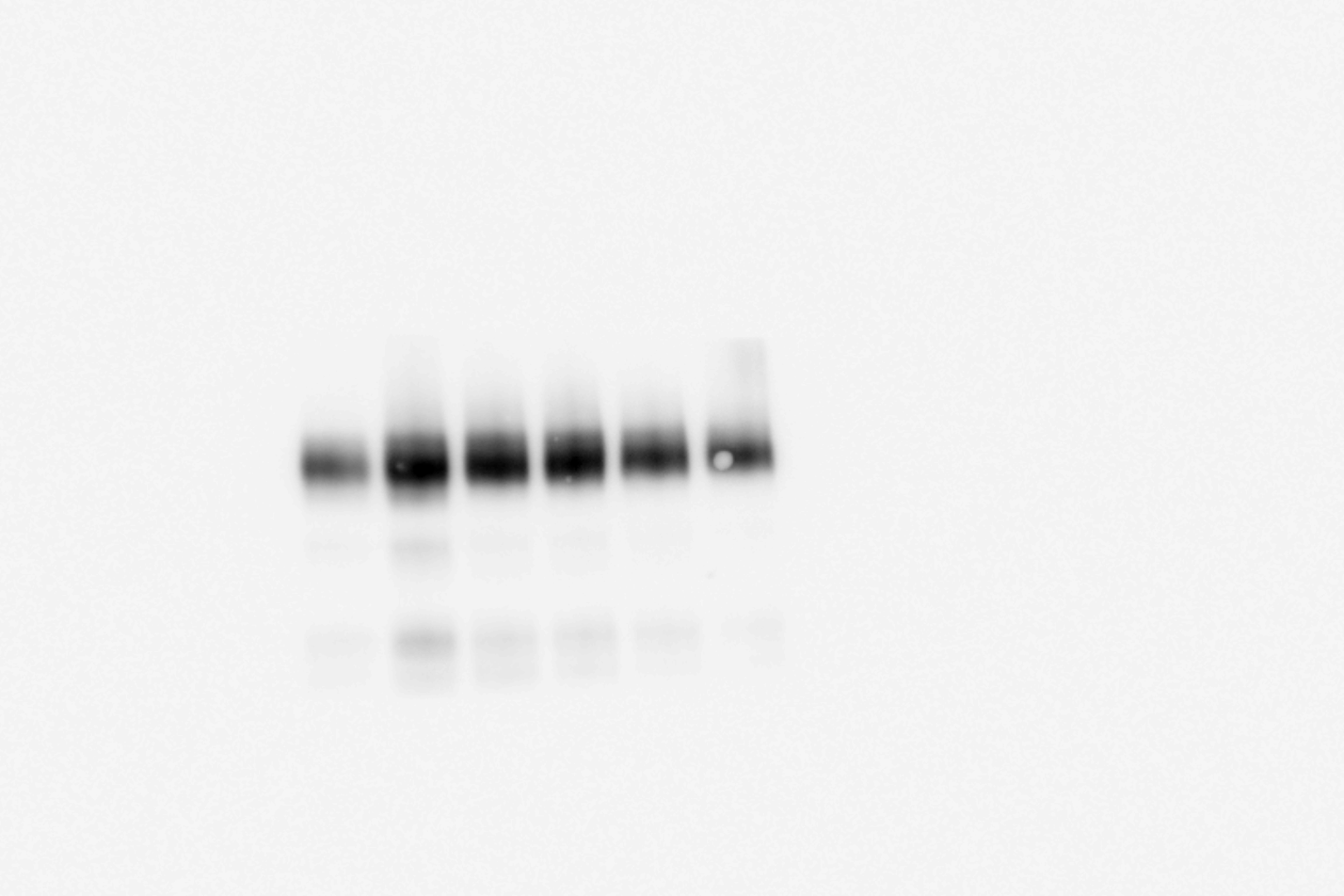 | **β-Actin**  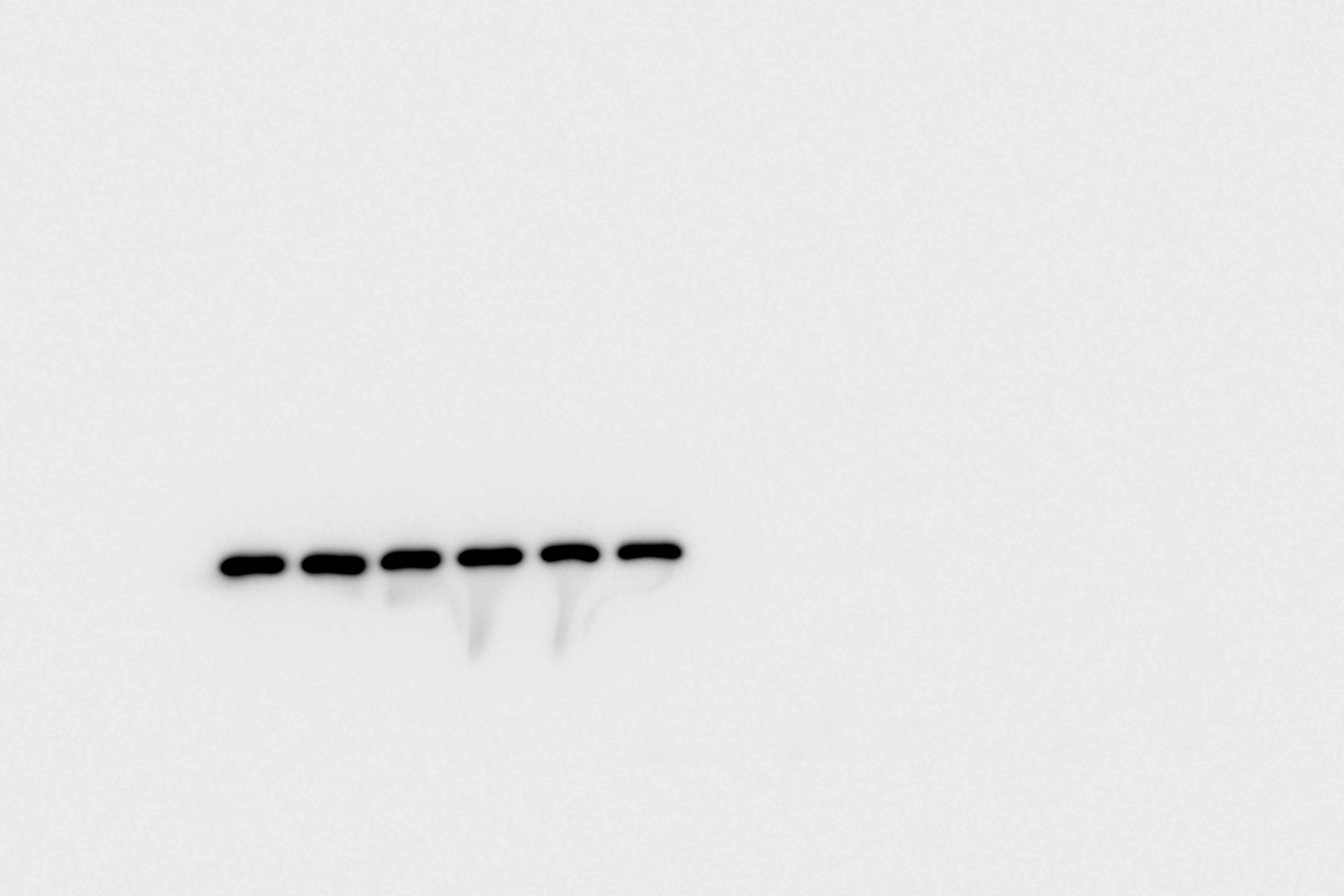 |
| --- | --- |

**(I)**

| **Fibronectin**  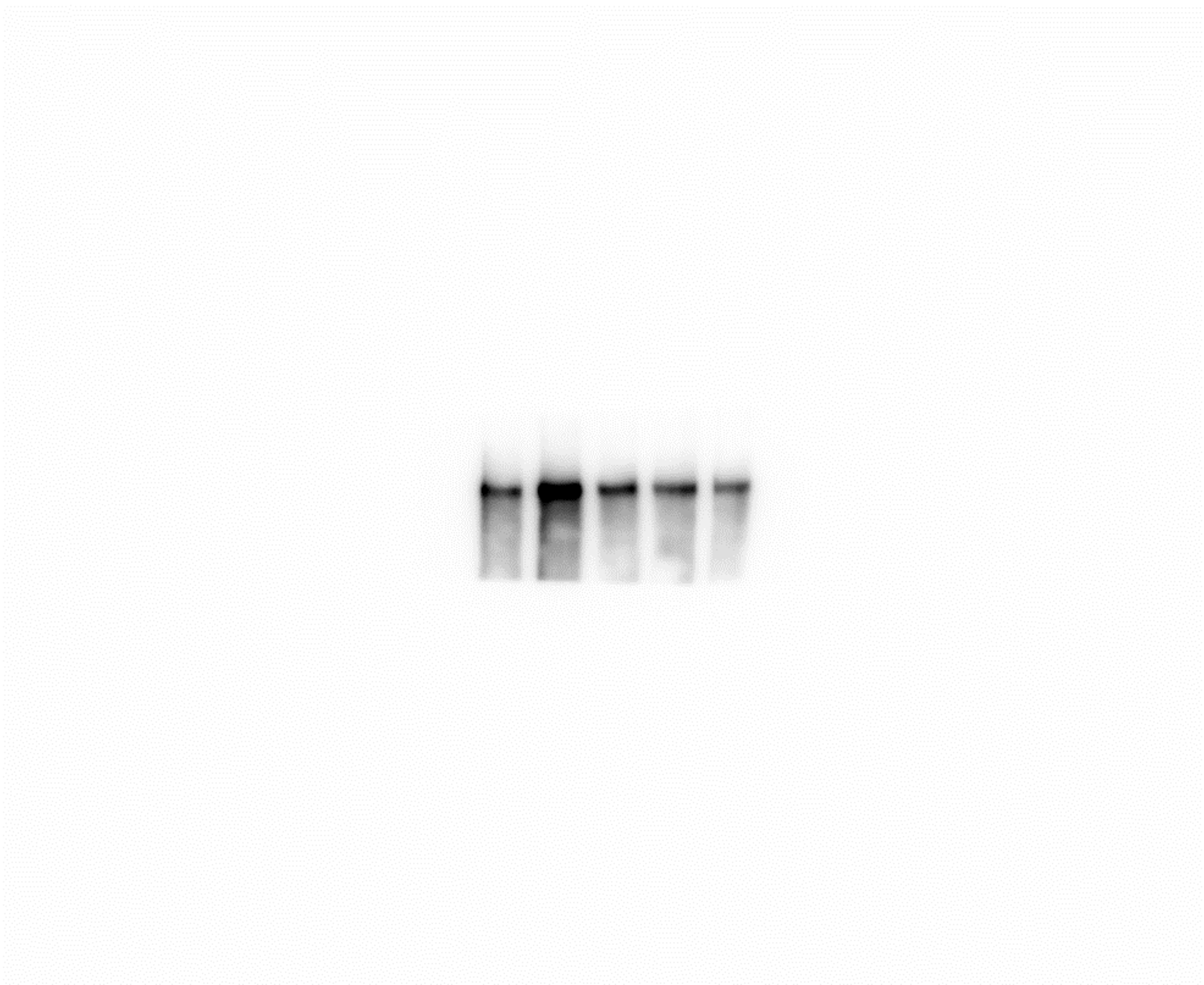 | **Periostin**  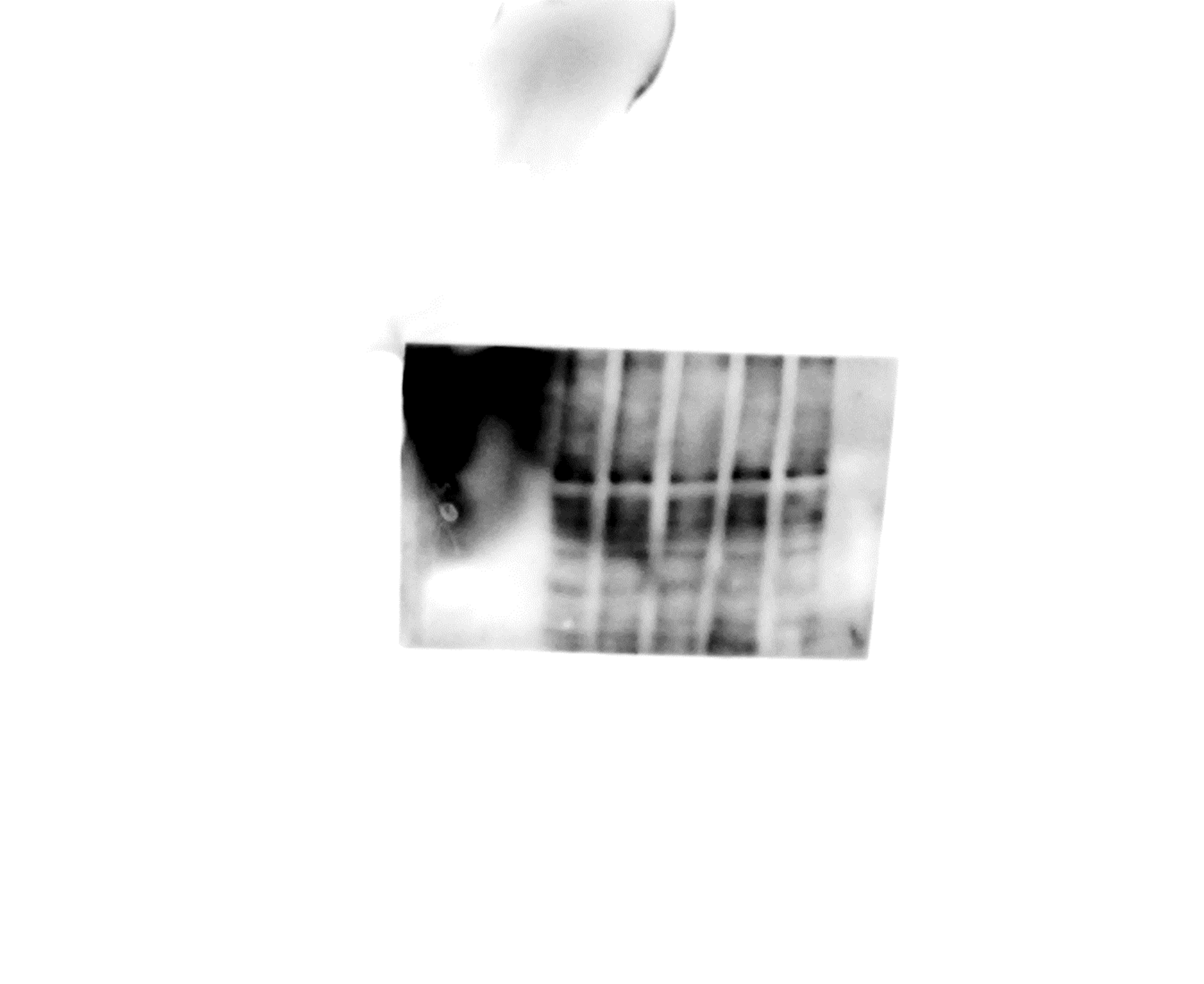 |
| --- | --- |

**(J)**

| **Collagen Type I-α1**  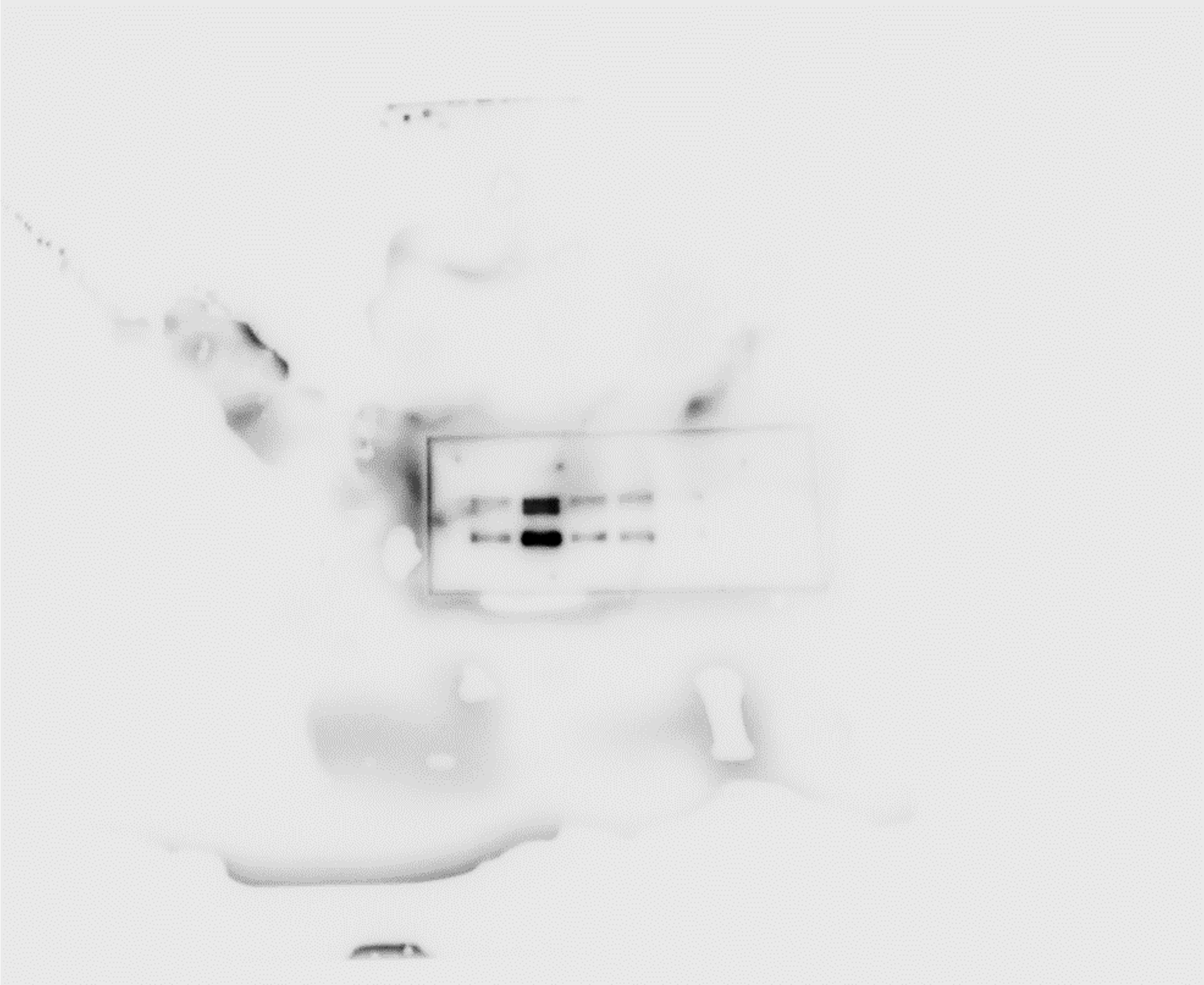 | **Integrin-α5**  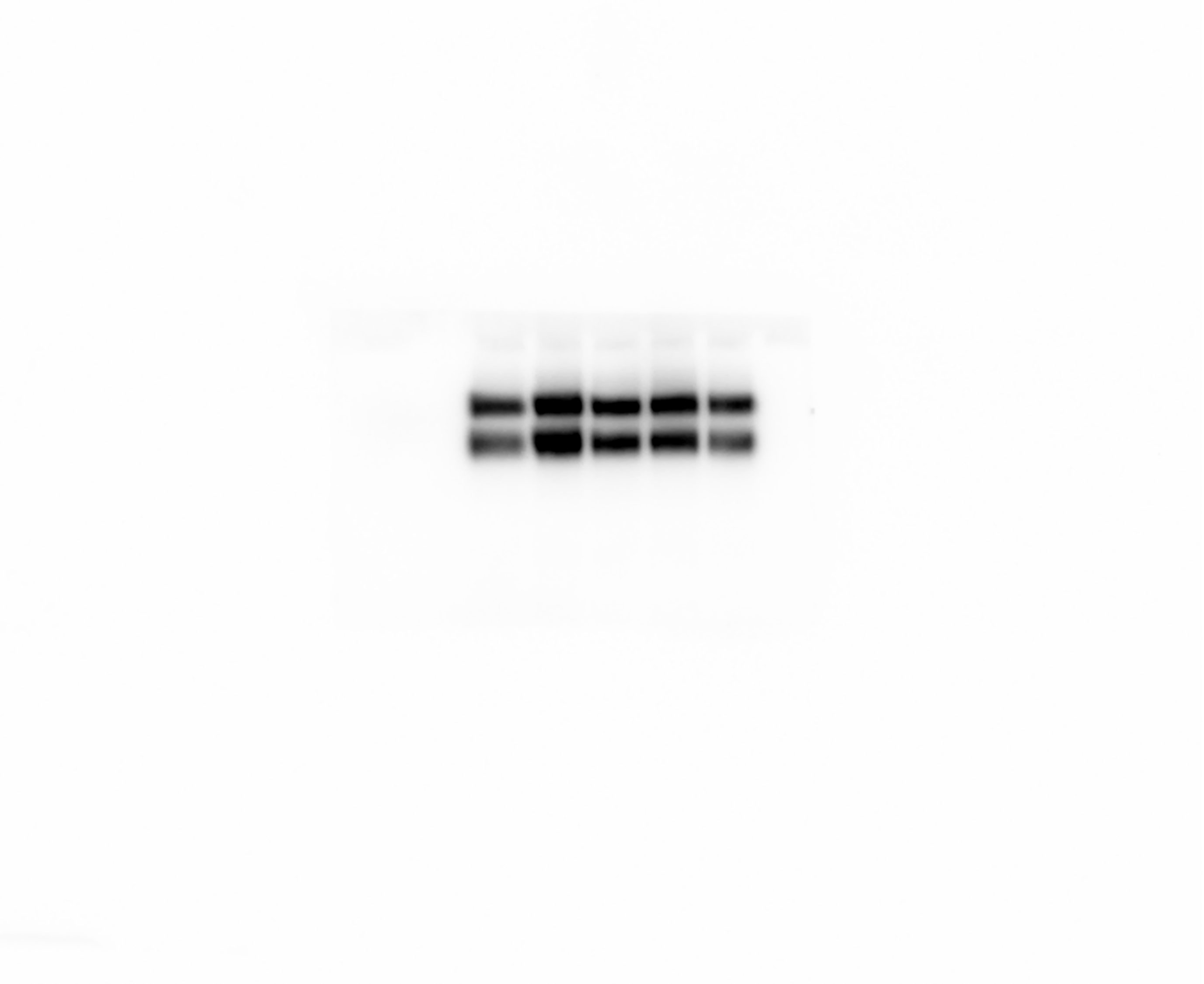 |
| --- | --- |

**(K)**

| **β-Actin**  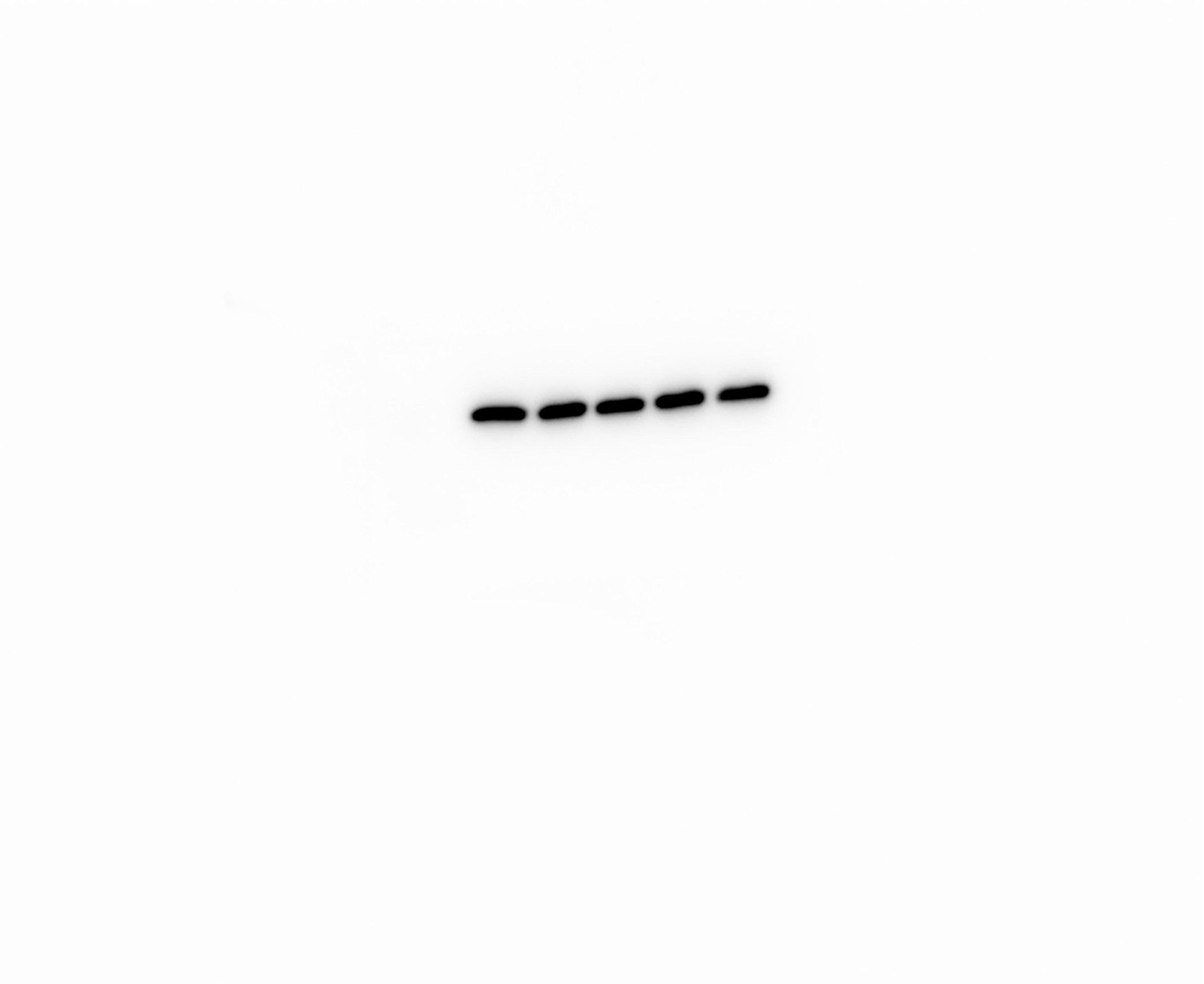 |  |
| --- | --- |

**Supplemental Figure 1. Original Western Blot images**

Original Western Blot images of Figure 1C (A,B), Figure 2A (C-E), Figure 3A (F-H), Figure 3C (I-K).


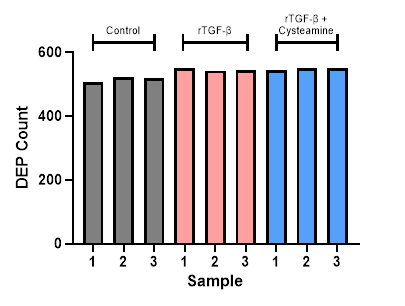


**Supplemental Figure 2. Quantification of differentially expressed proteins derived from EVs**

Bar graph illustrating the quantification of differentially expressed proteins in EVs identified via LC-MS/MS analysis of treated hPTECs.


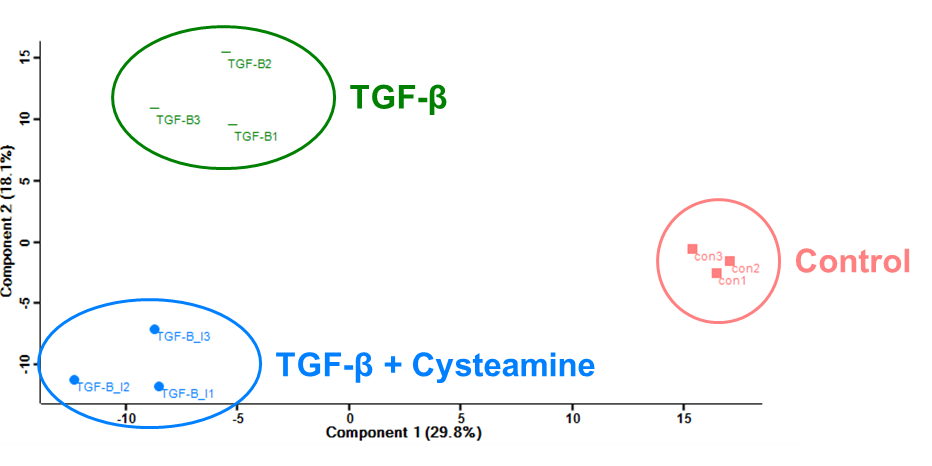


**Supplemental Figure 3. Principal component analysis of EVs from treated hPTECs.**

Principle component analysis plot of EV DEPs that show well-discriminated DEPs according to the treatment of TGF-β and cysteamine in LC-MS/MS analysis.


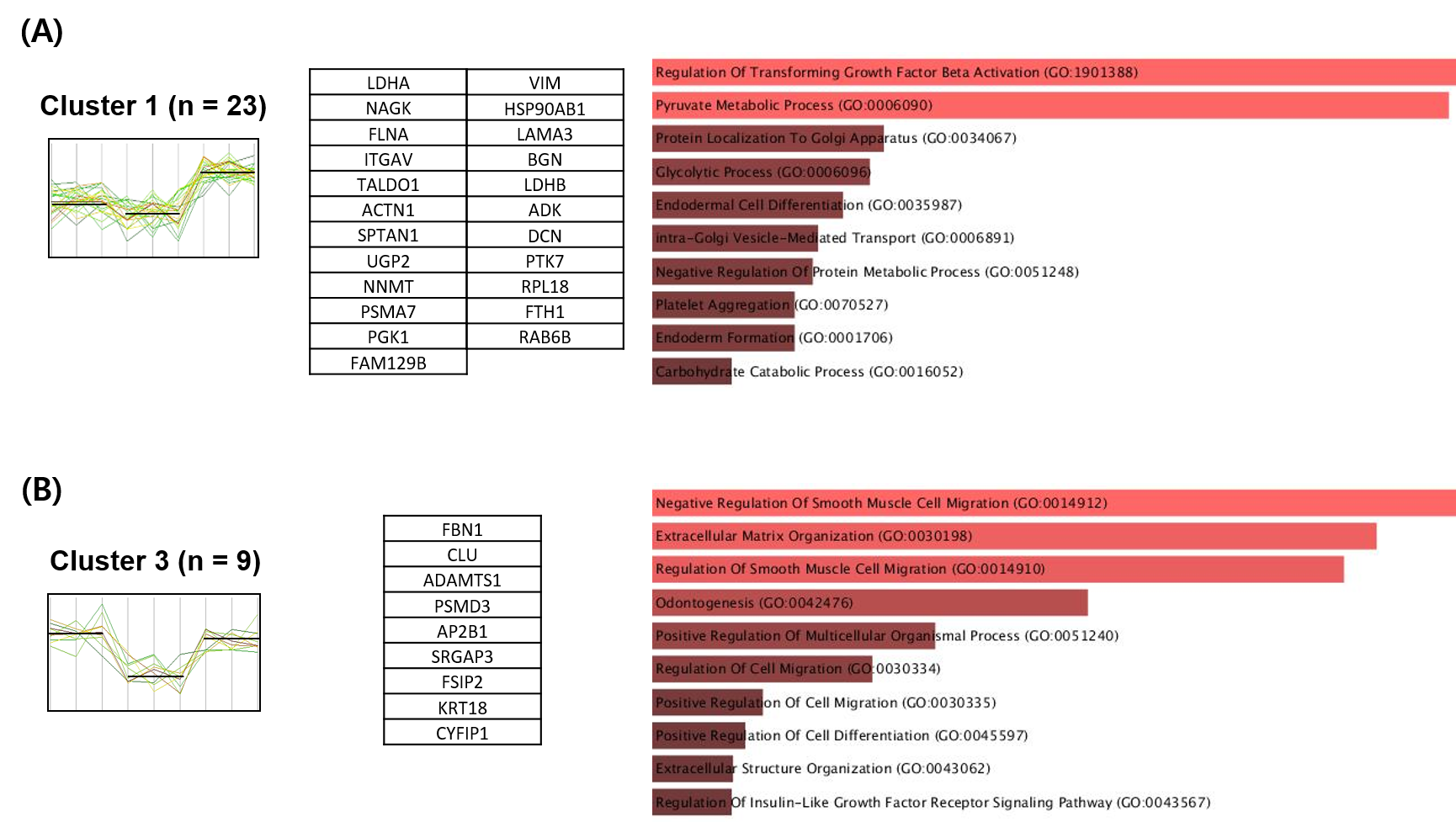


**
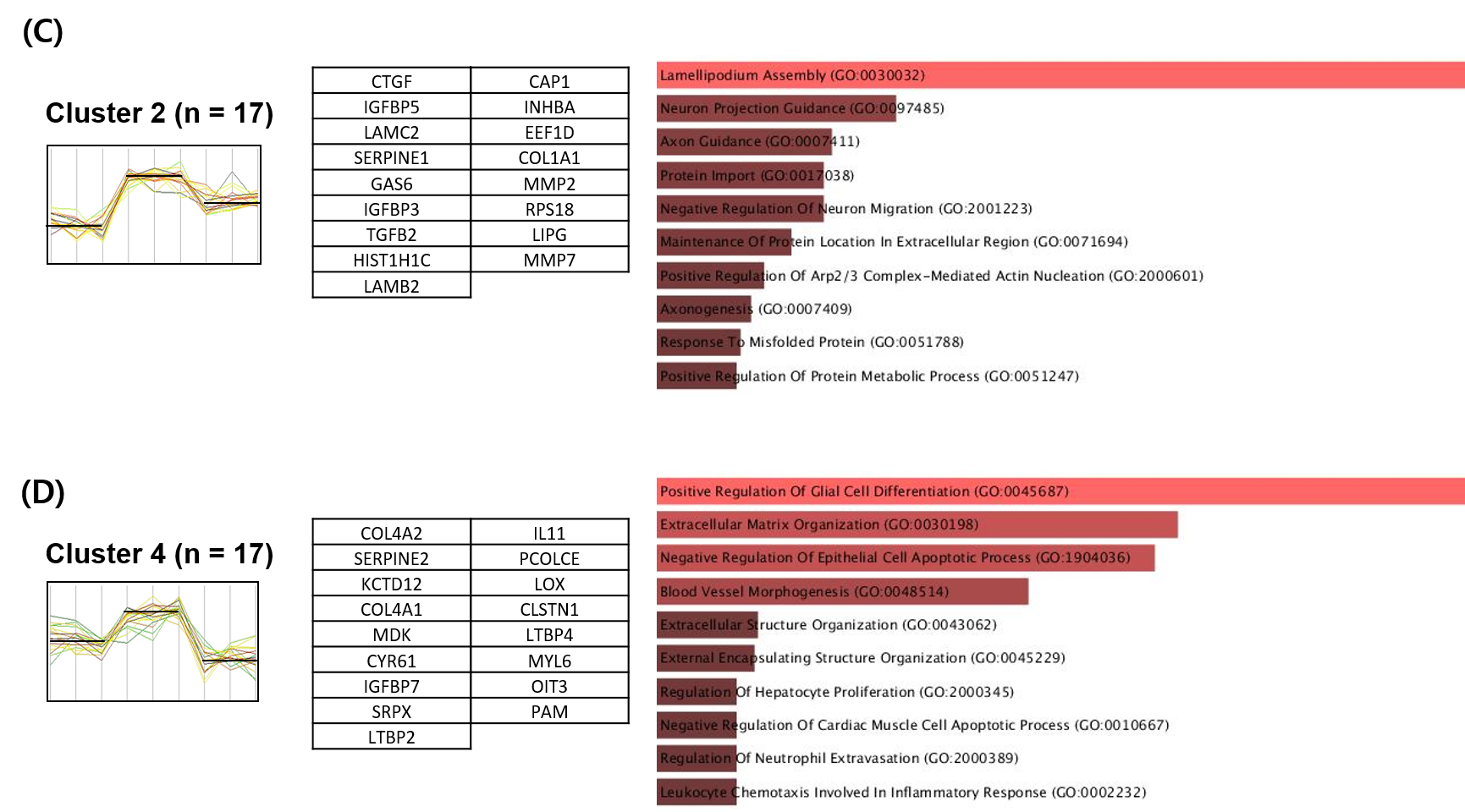
**

**Supplemental Figure 4. Cluster-specific functional enrichment analysis reveals distinct biological signatures**

Based on distinct patterns of intensity in response to the treatment conditions, four clusters were identified. The composition of differentially expressed proteins within these clusters is as follows: Cluster 1 comprises 23 DEPs, Cluster 2 consists of 17 DEPs, Cluster 3 encompasses 9 DEPs, and Cluster 4 includes 17 DEPs. Subsequent Enrichr analyses were conducted for each cluster, focusing on Gene Ontology (GO) Biological Process terms. The results are presented in order of ascending p-values, visually represented by the size of corresponding bars, where longer bars correspond to smaller p-values.
